# Structural insights from lipid-bilayer nanodiscs link α-Synuclein membrane-binding modes to amyloid fibril formation

**DOI:** 10.1038/s42003-018-0049-z

**Published:** 2018-05-03

**Authors:** Thibault Viennet, Michael M. Wördehoff, Boran Uluca, Chetan Poojari, Hamed Shaykhalishahi, Dieter Willbold, Birgit Strodel, Henrike Heise, Alexander K. Buell, Wolfgang Hoyer, Manuel Etzkorn

**Affiliations:** 1 Institute of Physical Biology, Heinrich-Heine-University, Universitätsstrasse 1, 40225 Düsseldorf, Germany; 2 Instititue of Complex Systems (ICS-6), Forschungszentrum Jülich, Wilhelm-Johnen-Strasse, 52428 Jülich, Germany; 3 Department of Physics, Tampere University of Technology, Korkeakoulunkatu 10, 33720 Tampere, Finland; 4 Department of Physics, University of Helsinki, Gustaf Hällströmin katu 2a, 00560 Helsinki, Finland

## Abstract

The protein α-Synuclein (αS) is linked to Parkinson’s disease through its abnormal aggregation, which is thought to involve cytosolic and membrane-bound forms of αS. Following previous studies using micelles and vesicles, we present a comprehensive study of αS interaction with phospholipid bilayer nanodiscs. Using a combination of NMR-spectroscopic, biophysical, and computational methods, we structurally and kinetically characterize αS interaction with different membrane discs in a quantitative and site-resolved way. We obtain global and residue-specific αS membrane affinities, and determine modulations of αS membrane binding due to αS acetylation, membrane plasticity, lipid charge density, and accessible membrane surface area, as well as the consequences of the different binding modes for αS amyloid fibril formation. Our results establish a structural and kinetic link between the observed dissimilar binding modes and either aggregation-inhibiting properties, largely unperturbed aggregation, or accelerated aggregation due to membrane-assisted fibril nucleation.

## Abstract (web summary)

Thibault Viennet and colleagues gain structural insight into amyloid fibril formation from their innovative use of lipid bilayer nanodiscs. This study connects α-Synuclein membrane binding modes to its aggregation properties, furthering our understanding of the cause of neurodegerative diseases.

## Introduction

The protein α-Synuclein (αS) is associated with various synucleinopathies including Parkinson’s disease through its abnormal aggregation, fibril formation, and formation of Lewy bodies^1–4^. Although its exact native function is not yet fully understood, αS is found in synaptic vesicles and supposed to be involved in membrane interactions, e.g., in synaptic vesicle homeostasis^5,6^ and SNARE-like vesicle-to-vesicle or vesicle-to-membrane fusion^7,8^. Membrane association of αS has been shown to modulate its aggregation propensity^9,10^ and αS oligomeric species have been proposed to be the toxic species in Parkinson’s disease, especially through membrane pore formation mechanisms^11,12^. Notably, αS has been shown to be specifically acetylated at its N-terminus, which is thought to act as an important mode of regulation of protein–membrane association^13,14^.

Previous data recorded using micelle and vesicle preparations already provided valuable information of the αS–membrane interactions, including binding and lipid specificity^15–18^, effect of mutations on membrane association^8,19^, micelle-bound structure^20^, vesicle-bound structural insights^21–23^, and conformational dynamics^8,23,24^. Two structural models of lipid-bound αS were proposed, i.e., the “extended helix” consisting of one roughly 100-residue-long α-helix^25^ and the “horse-shoe” consisting of two helices with different lipid affinities separated by a kink at residues 42–44^26^. Furthermore, various effects of lipids for αS aggregation were reported including inhibition of aggregation^27^, triggering of fibrillation^28,29^, and modification of fibril structure^29^. Membrane binding and its effect on aggregation have been shown to be strongly dependent on chemical properties of the lipids including head group charge content^26^ and fatty acid type^30^.

Although the phospholipid bilayer nanodisc (NDs) system^31^ does not fully resemble the physiological properties of synaptic vesicle membranes in all aspects (e.g., absence of curvature and integral membrane proteins), it offers the potential to provide additional insights that are complementary to the information obtained, e.g., on micelles or liposome preparations. Notably, NDs have been used before to study the effect of calcium ions on the membrane interaction of αS^32^, as well as lipid and monomer specificity of the Alzheimer-associated Aβ peptide^33^. In general, NDs are very homogeneous, stable in a wide buffer range^34^, and allow the preparations of well-defined lipid mixtures with an accurate estimate of the bilayer size^35^, charge^36^, and lipid molarity^37^. The increased stability may, e.g., offer the possibility to determine the interaction with a stable planar bilayer surface. In contrast, it is known for small unilamellar vesicles (SUVs) that the interaction with αS can considerably and rapidly change the lipid environment (e.g., from homogeneous SUVs to rather heterogeneous particles^8,38–40^). In addition, the smaller size of the NDs should, in theory, allow the detection of the lipid-bound state using suitable solution nuclear magnetic resonance (NMR) techniques^34,41,42^. In general, the well-defined size and lipid composition of NDs, paired with their accessibility, homogeneity, and stability should permit unique quantitative insights into αS membrane association and its role in aggregation.

Here we explore this potential and report on a comprehensive NMR investigation of the effects of lipid charge, bilayer fluidity, and αS N-terminal acetylation on the structural aspects of αS membrane association. We corroborate these insights with molecular dynamics (MD) simulations and a series of complementary biophysical measurements to further characterize membrane plasticity, global affinities, as well as binding and aggregation kinetics. Based on this data we correlate structural insights, such as residue-specific affinities and competition for accessible membrane surface area, to their potential role in modulating αS aggregation properties. Our study provides insights into (i) the different lipid binding modes of αS to stable planar bilayers of defined lipid quantity and composition, (ii) the effect of membrane plasticity for αS binding, (iii) the modulation of membrane plasticity through αS, and (iv) the connection between binding modes and their effect on αS aggregation. In addition, it gives an initial estimate of the number of lipid molecules and of lipid-associated αS molecules that are required to induce fibril nucleation, and allows to develop a basic structural and kinetic model of the modulation of αS aggregation through its interaction with different membrane surfaces. Our in vitro data help to better understand the molecular determinants of αS–membrane association and may point to possible in vivo implications in the context of Parkinson’s disease.

## Results

### αS membrane-modulated aggregation due to lipid charge

**Fig. 1:**
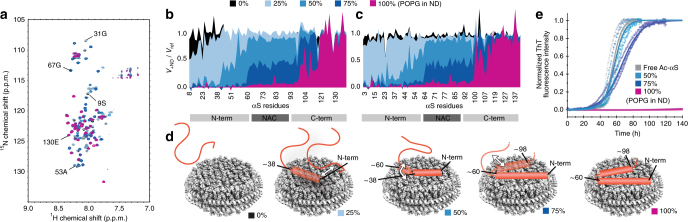
Lipid charge content modulates αS membrane binding modes and different binding modes show different effects on αS aggregation. **a** NMR [^15^N-^1^H]-TROSY-HSQC spectra of [^15^N]-αS (50 µM) in the absence (gray) or in the presence of 25 µM NDs containing an increasing amount of the anionic lipid POPG complemented with the zwitterionic lipid DMPC (0% POPG (black), 25% POPG (light blue), 50% POPG (blue), 75% POPG (dark blue), and 100% POPG (purple)). Selected residue assignments corresponding to differently affected parts of αS are indicated. Corresponding NMR attenuation profiles, i.e., the ratio of peak volumes in the presence and absence of NDs, are plotted against αS primary sequence for acetylated **b** and non-acetylated **c** αS. **d** Molecular model visualizing the gradual binding of different parts of αS to NDs with increasing charge content. White arrows and transparent coloring indicate αS regions that experience intermediate NMR-signal attenuation indicative of multiple (dynamic or static) states. **e** αS aggregation assays (normalized ThT fluorescence) in the absence and presence of NDs with indicated POPG content for acetylated αS (concentration of αS and NDs identical to NMR data in **a**–**c**, data of triplicate measurements until reaching saturation and their respective fits are shown, color code as in **a**–**c**)

To obtain residue-specific insights into the interaction of αS with lipid bilayer NDs of various composition, we recorded a series of solution NMR two-dimensional transverse relaxation optimized spectroscopy (TROSY)-heteronuclear single quantum coherence (HSQC) spectra keeping a molar ratio of one αS molecule per membrane leaflet (Fig. 1a). In the presence of NDs containing only 1,2-dimyristoyl-*sn*-glycero-3-phosphocholine (DMPC) lipids, no differences to the spectrum of αS in the absence of NDs were detected (Fig. 1a–c, black). This finding shows that αS does not interact with the membrane scaffold protein and provides additional evidence that αS does not interact with non-charged lipid bilayers. In a similar way as reported previously using liposomes^26,43^, we further tested the influence of increasing amounts of negatively charged lipid head groups on αS membrane association (Fig. 1a–c). It is noteworthy that lipid ratios and proper mixing of the different lipid types inside the NDs were also verified by NMR spectroscopy (Supplementary Fig. 1a). When increasing the content of the negatively charged lipid 1-palmitoyl-2-oleoyl-sn-glycero-3-phospho-(1′-rac-glycerol) (POPG) our NMR data show a gradually increasing bilayer interaction of αS, as evident by a residue-specific decrease in the ratio of NMR signals in the presence and absence of NDs. This NMR attenuation profile divides the protein into rather distinct regions with different membrane-binding behaviors (Fig. 1b, c). The first region spans the N-terminal residues 1–38, which are already weakly interacting at 25% content of negatively charged lipids and strongly interact at 50% (or higher) charge content. A comparison between αS that is acetylated at its N-terminus (Fig. 1b) and non-acetylated αS (Fig. 1c) shows that, in particular at lower anionic lipid content the N-terminus does interact stronger with the membrane when it is acetylated, which is in line with previous data observed on SUVs^14^. While we recorded most data for both αS forms, unless otherwise stated, only data obtained on the more physiologically relevant acetylated αS will be shown in the following. Data recorded on non-acetylated αS along with a more detailed discussion can be found in the Supplementary Information (Supplementary Fig. 2).

At 50% charge content, the region comprising residues 38–60 also gradually starts to interact. Amino acids 60–98, corresponding approximately to the aggregation-prone non-amyloid-β component (NAC region), display some interactions with membranes containing 75% anionic lipids and strongly interact at 100% anionic lipid content. The 98–120 region is (partly) affected by 100% net charge content only. Finally, the last 20 C-terminal residues never show any membrane interaction (see Fig. 1d for a model of the different binding modes). This data is largely in line with a predominantly electrostatic model^44^ (the first 60 residues displaying a net positive charge, the last 40 residues a net negative charge, and the NAC region being mostly hydrophobic), as well as the three regions dynamic model reported before using SUVs^23^.

Interestingly, a comparison with the previously published data on SUVs that observe similar negative charge-dependent binding modes for vesicle-bound αS using electron paramagnetic resonance (EPR) and NMR^26,45^, suggests that factors such as membrane curvature, potential instability of liposomes, or the physical borders introduced by the scaffold proteins, which should prevent formation of a fully extended αS α-helix on the membrane surface, do not have a large effect on the detected αS membrane association (see below for more detailed discussion on binding modes; also see Supplementary Note 1 for more detailed discussion on NDs stability).

Using Thioflavin T (ThT) fluorescence as a reporter for fibril formation, we also measured aggregation kinetics of αS in the absence and presence of the different ND compositions (Fig. 1e). These aggregation assays were recorded using identical protein and ND concentrations, as well as buffer conditions as used for the NMR measurements, facilitating a direct comparison between membrane-binding modes and their consequences for protein aggregation. It is noteworthy that, unless stated otherwise, an aggregation assay setup was chosen that mainly reports on the consequences of ND interactions with the lipid-independent aggregation pathway of αS^46–49^ (see Methods for more details).

Interestingly, despite the fact that the NMR data show interaction, the presence of NDs up to an anionic lipid content of 50% does not appear to affect aggregation kinetics of acetylated αS. When increasing the negative charge content to 75%, the aggregation half-time slightly increases (Fig. 1e, dark blue) and a strong aggregation-inhibiting effect is detected in the presence of NDs with 100% anionic lipids (Fig. 1e, purple). A comparison of the ThT kinetic data with the NMR-detected modes of αS binding to membranes of different charge contents allows to link molecular determinants of membrane association to their possible effects on αS aggregation. One of the most striking connections is that αS interaction with NDs comprising up to 50% negatively charged lipids does not involve the NAC region, and that under the same conditions no detectable effect on the aggregation behavior of (acetylated) αS is found in ThT assays. When further increasing the charge density above 50% negatively charged lipids, NMR data show first a partial (75% POPG, Fig. 1a–c, dark blue) and then a full (100% POPG, Fig. 1a–c, purple) signal attenuation of the NAC region. This membrane interaction of the NAC region correlates with a slight inhibitory effect of the 75% charged NDs on αS aggregation (Fig. 1e dark blue) and a very strong inhibitory effect of 100% charged NDs (Fig. 1e, purple). Therefore, our data strongly suggest that for the tested conditions (high anionic lipid content and high lipid-to-αS ratios) membrane association of the NAC region protects αS from aggregation.

### The ND-bound state of αS

**Fig. 2:**
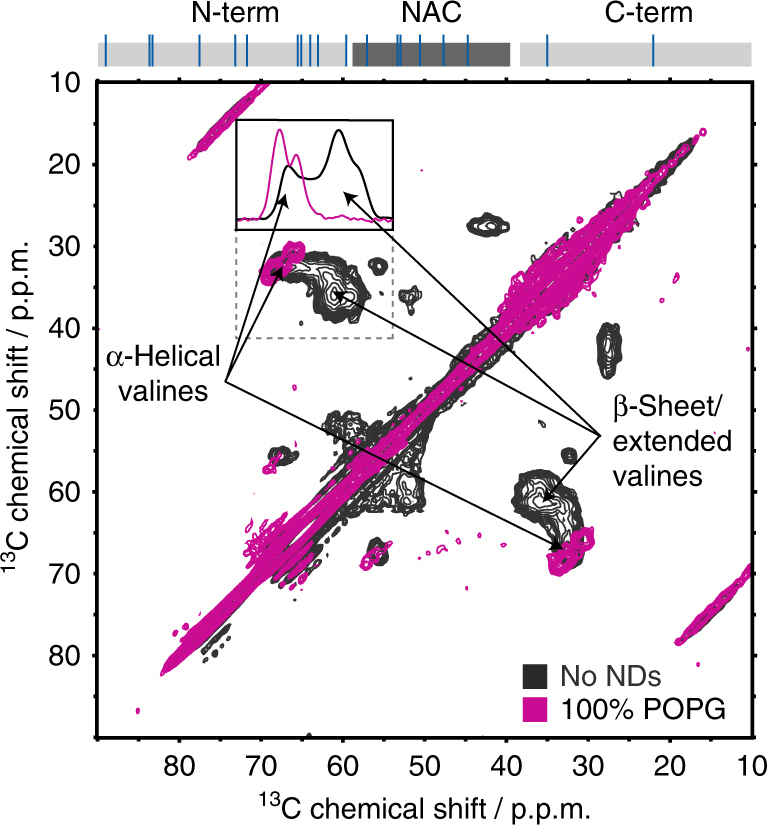
Nanodiscs binding induces α-helical structure in αS. [^13^C-^13^C]-Proton driven spin diffusion magic angle spinning-DNP spectra of free non-acetylated αS in frozen solution (black) and when bound to NDs with 100% POPG lipids (purple). Selective isotope labeling was used to specifically monitor valine Cα-Cβ chemical shift distributions. Peak positions indicative of β-sheet and α-helical secondary structure are labelled. The insert shows normalized 1D projections of the highlighted region (dashed square) in the absence (black) and presence of 100% POPG NDs. Signal deconvolution of these spectra reveals that about 92% of the valines are in an α-helical configuration in the presence of 100% POPG NDs. The occurrence of valine residues in the αS sequence is shown on top (blue lines). According to the respective solution NMR attenuation profile (Fig. 1c, purple) 18 out of 19 valines (i.e., 94.7%) are expected in the membrane-bound state at the used conditions

It is noteworthy that, despite possible in principle^34^, we could not detect the ND-bound conformation of αS by solution NMR (see Supplementary Fig. 3 and Supplementary Note 2 for more detailed discussion). In order to still gain insight into the conformation of αS bound to NDs, we used magic angle spinning solid-state NMR. Moreover, we took advantage of the very low temperatures (100 K) used in dynamic nuclear polarization (DNP) to additionally eliminate exchange processes and to increase the sensitivity of the experiment. To avoid problems of signal overlap arising from severe inhomogeneous line broadening often seen in this range of temperatures^50^, we used a sparse isotope labeling scheme^51^, leading to the simplification of ^13^C–^13^C spectra to secondary structure-sensitive Cα–Cβ cross-correlations of valines (and leucine Cβ–Cγ). Notably, according to the αS primary sequence and our solution NMR observations (Fig. 1a–c), 95% of the valine residues (i.e., 18 out of the 19) should be membrane bound at the used charge content (100%) and αS-to-ND ratio (1:2). Although in the absence of NDs the DNP ^13^C–^13^C spectrum shows a continuous distribution of the valine Cα–Cβ cross-peaks reflecting the carbon chemical shifts of the allowed Ramachandran space (expected for an intrinsically disordered protein such as αS, see Fig. 2, black), a very strong peak shift to a defined chemical shift range typical for α-helical structure is visible after addition of NDs (Fig. 2, purple). The DNP data thus show that αS binds the NDs in α-helical conformation corroborating previous studies using circular dichroism (CD) spectroscopy of vesicles, solution NMR spectroscopy of detergents micelles, and solid-state NMR spectroscopy of SUVs^20,23,29^. Interestingly, in contrast to SUVs the lipid bilayer of the NDs system does have a defined edge that can act as physical barrier for the αS membrane interactions. Geometrical considerations suggest that a fully extended α-helix with about 60 residues will completely span the diameter of one ND. Therefore, either the formation of a significantly bent helix (parallel to the membrane scaffold proteins) or at least one kink in the helix is compulsory for the largest observed ND-binding modes. The presence of rather defined steps in the NMR attenuation profiles for 50% and 75% POPG content (Fig. 1b, c) would be in line with the latter and could indicate possible kink positions (as visualized in Fig. 1d). The high similarity to the previously reported SUV-binding modes suggest that αS, in a similar manner as the so-called “horse-shoe” model^22^, has an intrinsic propensity to form the necessary kink in the membrane-binding interface.

### αS membrane-modulated aggregation due to lipid phase

**Fig. 3:**
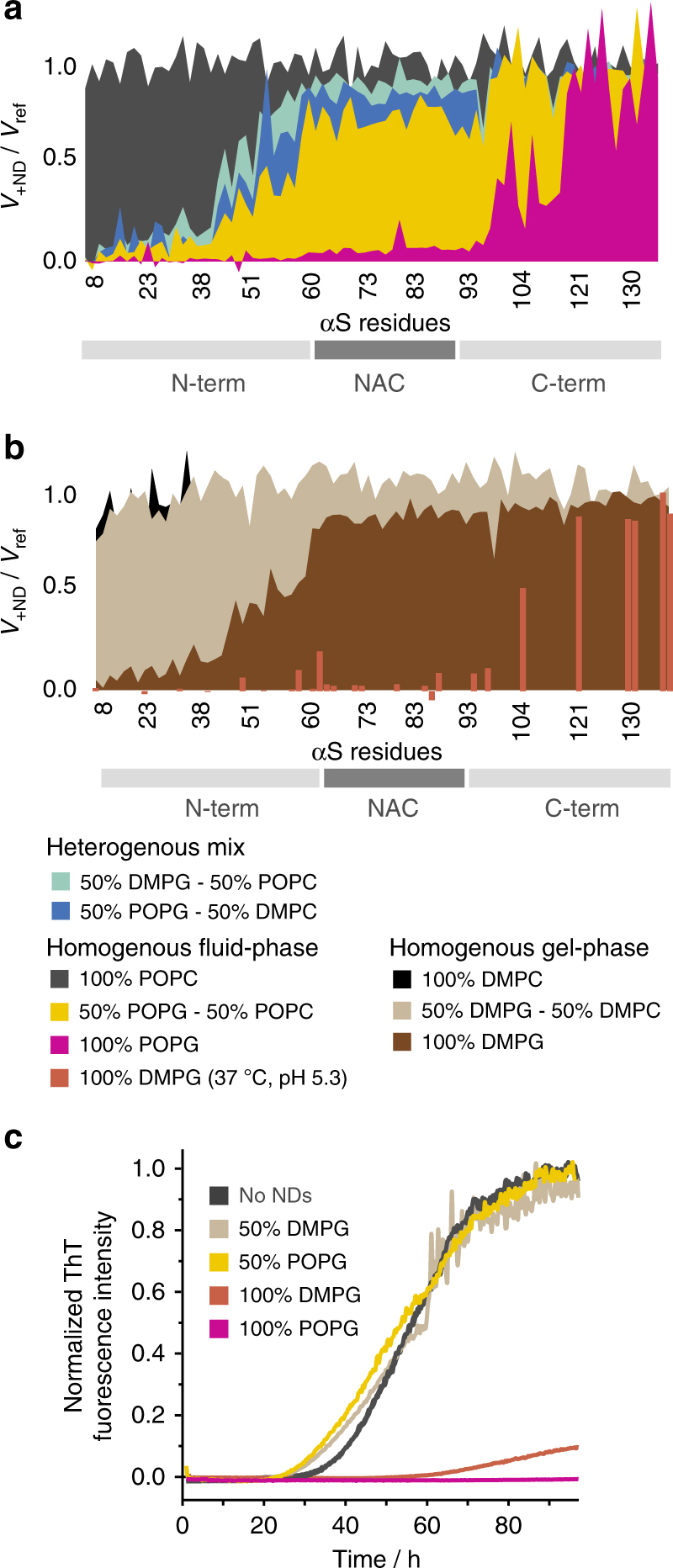
αS–lipid interaction is favored by increased membrane plasticity. [^15^N]-αS (50 µM) NMR attenuation profiles in the presence of 25 µM NDs with indicated lipid composition (molar ratio 2:1, αS-to-NDs) for bilayers that are in a more fluid **a** or gel phase **b** at 10 °C. It is noteworthy that all NMR data are recorded at 10 °C and pH 7.4 with the exception of one of the 100% DMPG samples (**b**, red bars) that was recorded at 37 °C and pH 5.3. **c** Corresponding ThT aggregation assays (50 µM αS, 25 µM NDs) for selected conditions (for better visibility only the mean values of triplicate measurements are shown, colors correspond to respective attenuation profiles in **a**, **b**)

To further investigate the effect of different lipid properties we recorded NMR spectra of αS in the presence of NDs containing different lipids and lipid mixtures. The data recorded with 100% 1-palmitoyl-2-oleoyl-sn-glycero-3-phosphocholine (POPC, one unsaturation, neutral charge) NDs do not show interaction (Fig. 3a, gray), comparable to 100% DMPC (no unsaturation, neutral charge) NDs (Fig. 1a–c, black). We also used all combinations of binary lipid mixtures of DMPC, 1,2-dimyristoyl-sn-glycero-3-phospho-(1′-rac-glycerol) (DMPG), POPC, and POPG, with an overall net charge content of 50%. Our data show that the heterogeneous mixtures DMPG/POPC and POPG/DMPC, and the homogeneous POPG/POPC mix behave very similarly (Fig. 3a), suggesting that the position of charge with respect to fatty acid as well as the ‘surface roughness,’ as potentially introduced by heterogeneous chain lengths, has little effect on αS–membrane interaction.

Interestingly, a mixture of fully saturated lipids (dimyristoyl lipids) with 50% charge content does not considerably interact with αS (Fig. 3b, beige). At the temperature of the NMR experiments (10 °C), the bilayer formed by saturated dimyristoyl lipids, in contrast to the partially unsaturated PO-lipids, is in the gel phase (*T*_m_ around 28 °C, Supplementary Fig. 1d). When increasing the charge content to 100%, but remaining in the gel phase (100% DMPG, Fig. 3b, brown), αS shows a clear interaction with the membrane resembling a binding mode that is found for 50% charge content in the fluid phase. This data is well in line with previous CD data on SUVs that identified an important role of the lipid phase for αS–lipid interaction^30^. Although increasing the temperature for the NMR measurements above *T*_m_ leads to a previously observed loss of NMR signals due to amide-water exchange processes for most relevant residues, lowering the pH from 7.4 to 5.3 can counter this effect (Supplementary Fig. 3c-f). The respective NMR data show that αS forms a much larger binding interface with DMPG lipids in the fluid phase (Fig. 3b, red-brown bars, only peaks with easily transferable resonance assignments are plotted), in line with previous findings using vesicles^30,44^, providing residue-specific insights into the modulation of αS membrane-binding modes by lipid phase properties.

Taken together, these data on αS–lipid association can be summarized as follows: (i) unsaturations in the hydrocarbon chains, leading to increased membrane fluidity, are not sufficient to induce binding; (ii) the presence of heterogeneity in fatty acid chains and the combination of charge and unsaturation on the same lipid molecule are not critical; and (iii) in addition to charge, a lipid phase state that introduces an increased membrane fluidity is important for binding.

We also performed ThT aggregation assays with NDs containing selected lipid mixtures as investigated by NMR. All mixtures that contain 50% negatively charged lipids, independently of acyl chain heterogeneity or charge position, show consistently unaffected aggregation behavior (Fig. 3c). However, αS aggregation is drastically impeded in the presence of 100% DMPG NDs (Fig. 3c, red-brown). As aggregation assays were measured at 37 °C, our NMR data (Fig. 3b, red-brown) show that αS is in a lipid-binding mode, which involves the NAC region and therefore is expected to inhibit aggregation.

### αS and membrane plasticity: a two-way street

**Fig. 4:**
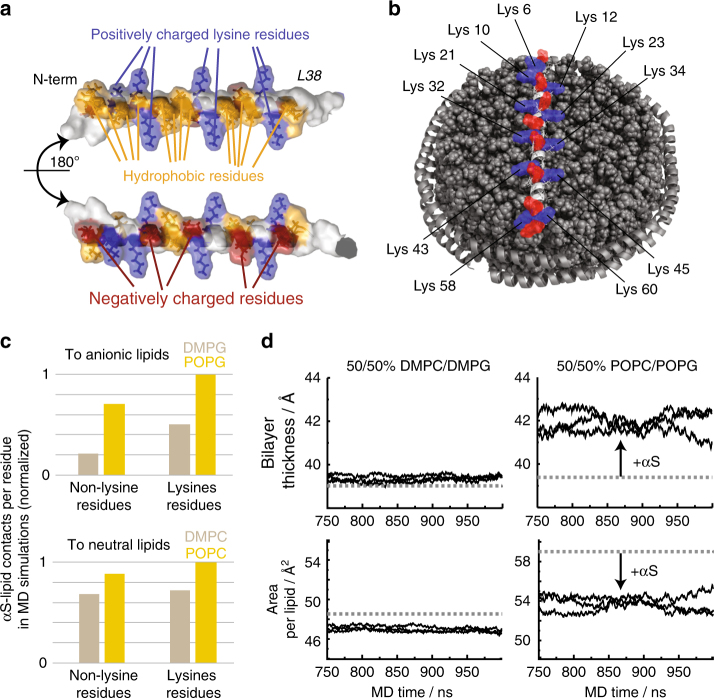
αS–lipid interaction potentially modulates membrane plasticity. **a** Molecular features of αS’s key lipid binding mode (residues 1–38), including periodically and symmetrically appearing lysine residues (blue) that form a positively charged “grid” (see text for more details). **b** Model of αS_1-61_-nanodisc interaction (drawn to scale), lysine (blue) and negatively charged residues (red) are highlighted. **c** αS-to-lipid contacts ( < 4 Å) per residue as occurring during the time course of MD simulations. Normalized values for interactions with anionic lipids (upper diagram) or neutral lipids (lower diagram) differentiating between lysines and all other residues as well as between gel/fluid-phase membranes (beige/yellow bars), respectively. **d** Bilayer thickness (upper panels) and area per lipid (lower panels) during the end of MD simulations in the presence (solid lines, three independent simulations) and in the absence of αS (dashed line). While simulations in the gel phase show no considerable effect (**d**, left panels), a clear trend toward a more ordered state for the fluid phase membrane, induced by the presence of αS, is visible by an increase bilayer thickness and reduced area per lipid (**d**, right panels)

It is apparent that the initial N-terminal αS residues comprise a central lipid-binding motif with key features highly suitable for interactions with a charged lipid surface^45,52^, and that these residues form a helical secondary structure after binding^16,23,29^. Based on the exposed and symmetric distribution of lysine residues, the occurrence of hydrophobic residues on one side of the helix and the distribution of negative charges on the opposite side (Fig. 4a), it is tempting to speculate that αS adopts a lipid-interacting conformation as shown in Fig. 4b. In this picture, it would be likely that the lipids and the lysine side chains (partly) rearrange, from their “unbound” conformation, to ideally accommodate electrostatic interactions. In line with our NMR results, this rearrangement may be favored by a more fluid lipid phase.

To test this hypothesis, we performed MD simulations of αS–membrane interactions. Our simulations focus on the first 61 residues of αS and their interactions with membranes formed by a mixture of either 50% POPG–50% POPC lipids in the fluid phase or a 50% DMPG–50% DMPC mix in the gel phase. The MD data confirm that lysine residues have a key role in the membrane interaction, as, e.g., visible by forming considerably more contacts to anionic lipids as compared with other residues (Fig. 4c, upper diagram). In addition, a generally stronger interaction of αS with the anionic lipids in the fluid membranes (POPG) is detected, as compared with the gel-phase membranes (DMPG) (Fig. 4c, yellow vs. beige). Noteworthy, these effects are much less pronounced for contacts to the neutral lipids (Fig. 4c, lower diagram). These findings correlate well with the effects of lipid charge and membrane plasticity seen in the NMR and aggregation assays.

Interestingly, the MD data also report on the effect of αS interaction from the lipid point of view. According to this data, the well-ordered DMPC/PG lipids (gel phase) experience very small effects due to the presence of αS. These MD results are in line with only small effects seen in differential scanning calorimetry profiles that we recorded on gel-phase NDs in the presence and absence of αS (see Supplementary Fig. 1e). On the other hand, for MD simulations of less ordered POPC/PG lipids (fluid phase), the presence of αS induces a considerably more ordered lipid state as evident by an increased bilayer thickness, reduced surface area per lipid, and increased order parameters for the hydrocarbon chains (Fig. 4d and Supplementary Fig. 4). In general, the MD data suggest that αS–membrane interaction is (initially) facilitated by increased membrane plasticity, e.g., via more contacts found in the fluid phase. These interactions may consequently confine lipids and lead to reduced membrane plasticity. The latter is in line with recent experimental data showing that αS binding can increase lipid packing^53,54^, an effect that has also been suggested to have a role in αS function as chaperone for SNARE-mediated vesicle fusion^55^.

### The role of affinities and kinetics

**Fig. 5:**
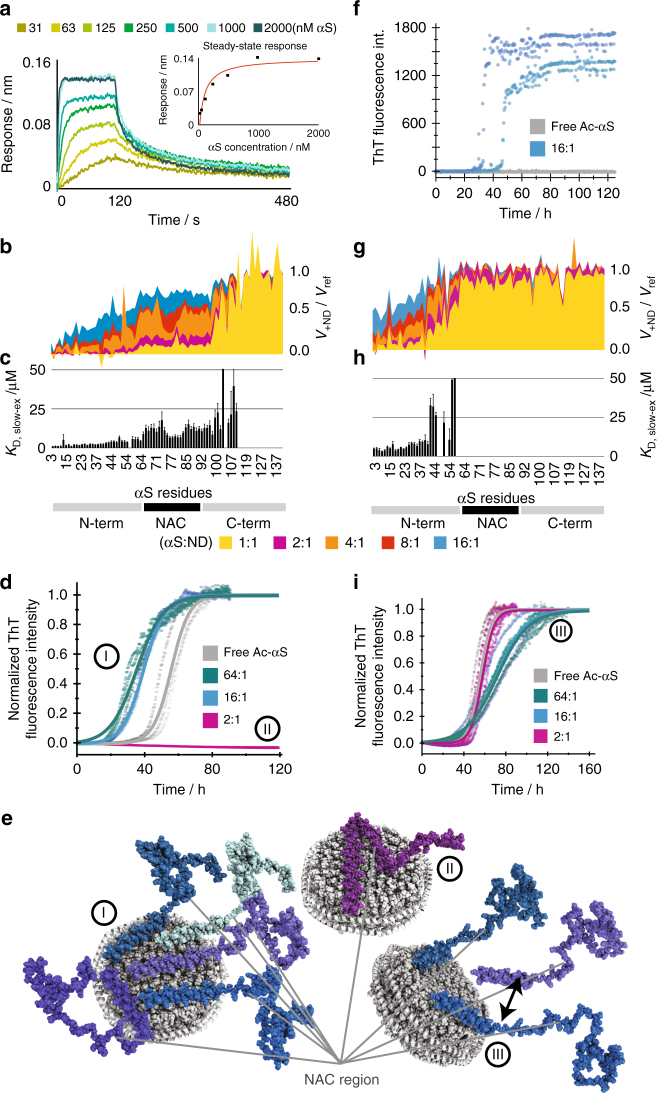
The interplay between interaction kinetics, differential residue specific affinities, membrane charge density and accessible surface area modulates αS aggregation. **a** BLI sensorgrams obtained with immobilized 100% POPG NDs and addition of different concentrations of αS. Corresponding steady-state response plot are shown as insert. A fitted global affinity (*K*_D_) of 67 ± 17 nM and a fitted off-rate of 0.015 ± 0.006 s^−1^ could be extracted. **b** NMR attenuation profiles of a titration of 50 µM αS with varying concentrations of 100% POPG NDs (αS-to-NDs molar ratios ranging from 16:1 to 1:1, see color code). **c** Corresponding residue-specific affinities extracted from NMR titration data. The values report on the slow-exchange (lower) limit for the affinities (see text for details). **d** Normalized ThT fluorescence aggregation curves for selected αS-to-ND ratios (conditions identical as in **b**–**g**). **e** NMR-derived binding modes and their link to the indicated aggregation behaviour (see Supplementary Note 3 for more details on how binding models were generated). Although high amounts of NDs with high charge density inhibit aggregation (binding mode II), limited amounts of highly charged membrane surfaces enhances aggregation (binding mode I). For NDs with a moderate lipid charged density, only one αS-binding mode was observed that has little effect on aggregation (binding mode III). **f** Nucleation ThT assays in quiescent conditions at pH 5.3. Although under these conditions no aggregation is observed in the absence of NDs (duplicates in gray), the presence of 16:1 molar ratio of 100% POPG NDs (duplicates in light and dark blue) induces primary nucleation. **g**–**i** Same as data shown in **b**–**d** but using NDs with 50% POPG content

In addition to modulation of binding modes due to lipid charge and membrane plasticity we were also interested in αS membrane-binding affinities and kinetics. We therefore measured interaction kinetics and thermodynamics using biolayer interferometry (BLI) with immobilized NDs of different charge contents. In line with the NMR data, no αS binding was detected when NDs containing 100% DMPC were immobilized. When NDs with 100% anionic lipid content were immobilized, a clear response upon addition of different αS concentrations was observed (Fig. 5a), enabling a quantitative description of the membrane association with an overall dissociation constants *K*_D_ of 67 ± 17 nM (one αS to one ND) and a slow off-rate of 0.015 ± 0.006 s^−1^.

In order to obtain residue-specific insights into the membrane affinity of αS, we additionally conducted NMR titration experiments using 100% negatively charged NDs (Fig. 5b). In general, affinities (*K*_D_) can be extracted from NMR titrations attenuation profiles by fitting the concentration dependency of the attenuation with a single exponential decay for each resolved peak (corresponding to one assigned residue in a two-state binding model, i.e., unbound and membrane attached). This method is valid under the assumption of a pure slow exchange regime. Although the BLI data clearly point to the presence of slow exchange processes, contributions from intermediate exchange are still to be expected for residues showing weaker membrane interactions, i.e., residues located in the central region of αS. For these residues, the applied method does not provide accurate quantitative values; nevertheless, a qualitative trend can still be extracted. It is noteworthy that the underestimation of exchange contributions will generate lower *K*_D_ values and hence the obtained values can be seen as a lower limit. The resulting slow-exchange-biased affinities (*K*_D,slow-ex_) are plotted in Fig. 5c and reveal differential membrane affinities for different regions of the αS primary sequence. As discussed above due to the slow-exchange bias, the differential affinities of αS are probably even larger. It is noteworthy that the regions with differential affinities for highly charged membranes largely overlap with the different binding modes induced by different charge densities identified before (Fig. 1b).

Due to the geometry of the used NDs, up to five αS molecules can simultaneously bind with a 38-residue long α-helix (first binding mode) to one side of one ND. If 8 molecules are accommodated together on the surface, (on average) a 23-residue long helix per monomer could be formed. The observed differential affinities are therefore a direct consequence of the competition of different monomers for accessible membrane surface area. As a result, membrane association of the weaker interacting NAC region is strongly dependent on the accessibility of negatively charged membrane surface. Importantly, it appears that one ND with 100% negatively charged lipids can simultaneously interact with about 16 αS molecules (8 per ND side) in the course of the NMR time scale, as seen from the complete disappearance of the signals of the very N-terminal residues (Fig. 5b, light blue). This means that under this condition the membrane surface brings several αS molecules, with nearly fully exposed NAC regions, in close spatial proximity.

To characterize the effect of the accessible membrane surface area on αS aggregation behavior, we measured ThT aggregation kinetics on samples with different αS-to-ND ratios by decreasing the ND concentration at constant αS concentrations (Fig. 5d). Interestingly, a higher ratio of αS-to-ND leads to a prominent decrease in aggregation lag times when using 100% POPG NDs (Fig. 5d, blue and cyan). These data show, in line with previously reported behavior on SUVs^28,29^, that under specific conditions lipid bilayers can accelerate the fibrillation process. Our NMR data allow to link these conditions, i.e., limited membrane surface area with a high charge density, to an αS–lipid-binding mode that brings several αS molecules with exposed NAC regions in close proximity (binding mode I in Fig. 5e).

In order to disentangle the effect of NDs on the nucleation or the elongation step in the αS aggregation pathway, we used ThT assays predominantly reporting on the one or the other (see methods for more details of assay design). Nucleation-sensitive assays in the presence of 100% POPG NDs and an αS-to-ND ratio of 16:1 indeed show that the underlying membrane association (binding mode I in Fig. 5e) enhances primary nucleation (Fig. 5f, blue). Interestingly, as our data also allow an estimation of the total number of αS monomers that are brought in close proximity due to their interaction with the same ND (i.e., up to 8 monomers per bilayer side, Fig. 5b blue), this result may also provide a first approximation of the number of αS monomers needed for the formation of a nucleus. As discussed above, our data suggest that this “minimal critical nucleation number” has an upper limit of around 8 αS molecules. It is noteworthy that this number is only an initial estimate and may be influenced by dynamic processes as well as local fluctuations, which may lower or increase the value by a few monomers.

Fibril elongation-sensitive aggregation assays carried out in the presence of 100% POPG NDs (Supplementary Fig. 5c, d) show no effect on elongation rates for αS-to-ND ratios of 16:1 (binding mode I in Fig. 5e). However, a clear reduction in elongation rates with decreasing αS-to-ND ratio is visible, which is largely in line with sequestering monomers, in particular accessible NAC region, out of solution (Supplementary Fig. 5c, d). No fibril elongation is observed for αS with a fully membrane-bound NAC region (binding mode II in Fig. 5e) consistent with the overall aggregation-inhibiting properties of this condition. Noteworthy, unlike in the case of SUVs^29^, atomic force microscopy (AFM) images of αS fibrils formed in the absence or presence of NDs do not show different morphology (Supplementary Fig. 6). This, however, does not exclude that (a limited amount of) lipids are also incorporated into the fibrils (see Supplementary Fig. 6 and stability considerations for more detailed discussion).

We additionally carried out the same BLI measurements, NMR titrations, and ThT assays for ND containing only 50% POPG lipids.

For these NDs, no clear signature of binding could be obtained in the BLI measurements, suggesting a weak affinity and/or too fast off rates to allow detection via BLI. This is in line with size exclusion chromatography (SEC) profiles that also point to a more transient interaction (Supplementary Fig. 1b, c).

NMR titrations, however, show clear concentration-dependent attenuation profiles that allow the calculation of (slow-exchange-biased) residue-specific affinities (Fig. 5g, h). Noteworthy, the NMR attenuation profiles and affinities for the αS residues in the first binding region (residues 1–38) are comparable to the values obtained for 100% charged NDs (Fig. 5b, c). In contrast, for the following binding regions much lower affinities are found (at the edge of detection for residues 39–60 and no interaction for residues > 60), including the absence of interactions of the NAC region. It is noteworthy that for the NDs with 50% anionic lipids the protein-to-ND ratio does not affect the overall binding mode (binding mode III in Fig. 5e). In line with an exposed NAC region, the ThT data for these NDs at low αS-to-ND ratios are consistently showing no effect on aggregation half-times (Fig. 5i). The data at higher ratios are less reproducible and show a slight tendency to prolonged elongation rates. Unlike for 100% POPG NDs and in line with the previously discussed moderate effects of 50% POPG NDs on the overall aggregation process, we did not observe accelerated αS nucleation in the presence of NDs with 50% POPG nor a clear perturbation of fibril elongation in seeded experiments (see Supplementary Fig. 7 for data and a more detailed discussion).

## Discussion

Overall, our data demonstrate that the ND system allows to study the interaction of αS with stable, planar membranes in a quantitative, and site-resolved way. Many aspects of the membrane association are similar to previously reported interactions with micelles or SUVs, suggesting that features that distinguish the different membrane mimetics, such as curvature, physical bilayer borders, and potential vesicle disruption do not substantially alter the membrane binding properties of αS. The convenient handling, reliable, and unique quantification properties, as well as the wide compatibility of the NDs systems for a broad range of biophysical techniques also enabled us to obtain a direct correlation between the structural and kinetic properties of the different membrane-binding modes and their consequences for αS amyloid fibril formation. In summary, our data show that (i) the N-terminal αS region interacts rather similarly with NDs composed of 100% or 50% anionic lipids; (ii) for 100% anionic lipids, the αS can adopt a substantially expanded binding mode as compared with 50% anionic lipid content, leading to considerably higher global affinities; (iii) the exchange rate between free αS in solution and membrane-bound αS is slow in the 100% charged case and likely to be faster in the 50% case; (iv) region-specific membrane affinities (especially the NAC region) are correlated with aggregation properties; (v) with sufficient excess of lipids and sufficient charge density, NDs can inhibit primary nucleation and fibril elongation by sequestering monomers out of solution; (vi) competition of αS monomers for highly charged lipid surface generates a membrane-bound αS conformation that can induce primary nucleation; and (vii) the number of αS monomers that are brought together by one ND and which can promote amyloid fibril nucleation is in the order of about 8 αS molecules.

**Fig. 6:**
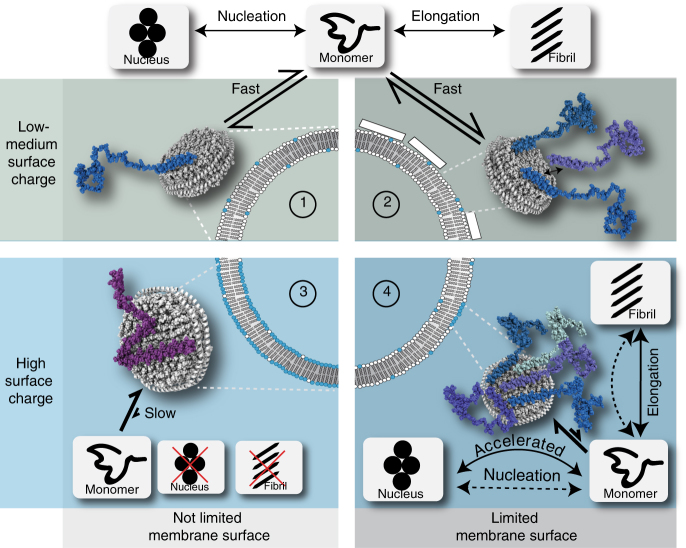
Model of the influence of different NDs on the αS aggregation pathways and speculations regarding their potential implication in the context of cell/vesicle membranes. Four scenarios are depicted summarizing our in vitro data in respect to membrane surface charge and accessibility. (Note that schemes with nanodiscs and aggregation pathways are based on our findings, while possible implications in respect to physiological membranes are purely speculative.) In cases where membranes/NDs with only low charge densities are present (scenario 1 and 2) αS interacts with its N-terminal residues and forms an exchanging equilibrium between soluble and membrane associated αS monomers (binding mode III in Fig. 5e). This equilibrium does not seem to strongly interfere with the slow process of αS nucleation, it may however (slightly) decrease the pool of free monomers available for fibril formation. Although physiological membranes, in general, have a lower average charge density (represented by the upper panels), specific, abnormal, and/or stochastic processes may also lead to highly charged lipid clusters (lower panels). In the unlikely case of not limiting surface access (scenario 3) αS will interact in a binding mode that will largely inhibit both αS nucleation and fibril elongation (binding mode II in Fig. 5i). In cases with only local charge clusters, several αS monomers may compete for the limited highly charged membrane surface area (scenario 4). This binding mode (Fig. 5e mode I) can bring exposed NAC regions of several αS monomers in close proximity and accelerate the amyloid fibril nucleation process

Although our in vitro data directly support the above drawn conclusions, we can only speculate about which (if any) role these factors may have in vivo. Figure 6 summarizes the experimentally determined molecular and kinetic determinants of membrane-modulated αS aggregation and speculates about their potential physiological roles. In general, it should be noted that, in our study, the strongest effects were observed at lipid charge densities well above the average lipid compositions of native membranes. However, the normally found high lipid diffusion rates in physiological membranes may generate clusters of higher negative charges that may form spontaneously or be induced by an initially transient αS interaction. For the latter, the N-terminal acetylation may have an important role, as it increases membrane interaction at native lipid charge densities. Our data suggest that clusters of around 60–80 negatively charged lipids suffice to form a strong interaction (this may however not be the lower limit and specific physiological lipids not tested here may have even stronger effects). Sporadically formed charged lipid clusters could also induce a competition of several αS monomers for the accessible surface area. Our data show that due to the different residue-specific membrane affinities, this will generate a binding mode that, once the rather low αS critical oligomerization number is reached, can act as an aggregation seed. Such a scenario could promote the initial step of primary nucleation in the pathogenesis of Parkinson’s disease and is in line with recent in vivo findings, suggesting that shielding αS from membrane interactions can inhibit initial steps of amyloid fibril formation including the formation of cell-toxic species^56^.

## Methods

### αS expression and purification

Codon-optimized αS in the pT7-7 vector was expressed in *Escherichia coli* BL21 DE3. For acetylated αS, the N-terminal acetylation enzyme NatB from *Schizosaccharomyces pombe*, which will selectively acetylate αS at the free amino group of the N-terminus, was coexpressed in a second vector, pNatB^57^. Expression was conducted in 50 mM phosphate-buffered 2YT-medium (pH 7.2) with 0.4% glycerol and 2 mM MgCl_2_, protein production was induced at OD 1-1.2 with 1 mM Isopropyl β-D-1-thiogalactopyranoside (IPTG) and run for 4 h at 37 °C. For ^15^N-labeled protein, αS or acetylated αS was expressed in M9 medium with 0.2% ^15^NH_4_Cl.

Sparsely labeled αS for DNP experiments was non-acetylated, expression was done in a similar way, in M9 medium using 0.4% [2-^13^C]-glucose and 0.2% ^15^NH_4_Cl. Isotope labeling of Phe, Gln, Glu, Pro, Asn, Asp, Met, Thr, Lys, and Ile was suppressed by supplementing sufficient quantities (150 µg ml^–1^ of each) of these unlabeled amino acids in the expression media as reported previously^51^.

Purification of αS or acetylated αS was carried out as previously described^58^, some changes to the original protocol have been made. Except for sparse labeled αS for which previous lysis in 20 mM Tris-HCl pH 8.0, 1 mM EDTA was done, cell lysis and release of αS or acetylated αS was performed by directly boiling the frozen cell pellet at 95 °C in a threefold volume of 20 mM sodium phosphate buffer, pH 7.4, for 30 min. Thermostable αS or acetylated αS remained in the supernatant after 30 min of centrifugation at 15,000 × *g* and 4 °C and was subsequently subjected to an ammonium sulfate precipitation by slowly adding saturated ammonium sulfate solution to 50% saturation at 4 °C. Precipitated protein was pelleted at 15,000 × *g* and 4 °C, dissolved in 50 ml of 50 mM Tris-HCl pH 8, sterile-filtered, and loaded onto an ion exchange chromatography column (HiTrap Q FF, GE Healthcare), where αS or acetylated αS eluted at around 300 mM NaCl in 50 mM Tris-HCl pH 8. Elution fractions containing αS or acetylated αS were subjected to another ammonium sulfate precipitation and finally purified by a SEC run (Superdex 75 10/300, GE Healthcare) in 20 mM sodium phosphate pH 7.4, 50 mM NaCl.

N-terminal acetylation of acetyl-αS was checked by high-performance liquid chromatography, mass spectrometry, and NMR, proved to be about 95% when co-expressed with NatB.

### Membrane scaffold protein expression and purification

As reported before^59^, *E. coli* BL21 (DE3) were transformed with MSP1D1 or MSP1D1∆H5 plasmid DNA in vector pET28a. Cells were grown in lysogeny broth (LB) medium, induced by 1 mM IPTG at an optical density of 0.7, and incubated 5–6 h at 37 °C, then pelleted down. Cells were resuspended in 50 mM Tris-HCl pH 8, 500 mM NaCl (buffer B) supplemented with 6 M GdnHCl and EDTA-free Complete protease inhibitors (Macherey–Nagel) lysed by sonication (Bandelin Sonopuls MS72 probe), centrifuged at 17,000 × *g* for 1 h (Beckman J2-21 rotor JA-20.1) and incubated 1 h with previously equilibrated 2.5 ml Ni-NTA agarose resin/3 l culture (Macherey–Nagel). Column was washed with 4 column volumes (CV) buffer B, 4 CV buffer B supplemented with 1% Triton X-100, 4 CV buffer B + 60 mM Na-cholate, 4 CV buffer B, and 4 CV buffer B + 20 mM imidazole. Four fractions of 1 CV were eluted with 250 mM imidazole. The whole process was kept at 4 °C in a cold room. The elution fractions were pooled and dialysed against 100-fold 200 mM Tris-HCl pH 7.5, 100 mM NaCl. N-terminal His-tag was cleaved using tobacco etch virus (TEV) protease incubated overnight at 4 °C. ΔHis-MSP was separated by immobilized metal affinity chromatography (IMAC) and concentrated to the desired molarity using a Vivaspin centrifugal device of 10 kDa molecular weight cutoff (MWCO).

### ND assembly

NDs were assembled according to established protocols^31^. In short, lipids’ chloroform stocks were dried under nitrogen flow to obtain a lipid film and stored under vacuum overnight. ΔHis-MSP1D1 or MSP1D1∆H5 and the appropriate amount of lipids (Avanti Polar Lipids) solubilized in 60 mM Na-cholate were mixed together in 20 mM Tris-HCl pH 7.5, 100 mM NaCl, 5 mM EDTA. The scaffold-to-lipids molar ratio was calculated from geometrical considerations. 20% w/v of previously washed Biobeads SM-2 (Biorad) were added and the mixture incubated at room temperature overnight. The Biobeads were removed by centrifugation and once again 20% w/v were added for an additional 4–5 h. Finally, they were purified by SEC on a HiLoad 16/600 Superdex 200 pg column (GE Healthcare) equilibrated with 20 mM sodium phosphate pH 7.4, 50 mM NaCl using a Äkta pure device at a flow rate of 1 ml min^–^^1^. The quality of NDs preparation was check by the SEC chromatogram and by DLS (PSS Nicomp). NDs were concentrated to the desired molarity using a Vivaspin centrifugal device of 10 kDa MWCO. It is noteworthy that concentration of NDs can be rather accurately determined using the 280 nm absorbance of the membrane scaffold proteins and lipid concentration can be estimated using the geometrically ideal lipid amount per ND, i.e., 156 DMPC molecules or 150 POPG molecules.

### Biolayer interferometry

NDs were immobilized on the sensor surface of amine reactive biosensors (AG2R) (fortéBIO, PALL Life Science) after 1-ethyl-3-(3-dimethylaminopropyl)carbodiimide/N-hydroxysulfoxuccinimide (EDC/NHS) activation to a final level between 1.2 and 1.8 nm depending on the NDs type using an Octet RED96 instrument (fortéBIO, PALL Life Science). All biosensors were quenched with 1 M ethanolamine for 3 min. All experiments were carried out in multi cycle kinetics at 25 °C. Association of αS in running buffer (20 mM sodium phosphate pH 7.4, 50 mM NaCl) on NDs and reference biosensors was recorded for 120 s, followed by a dissociation phase of 360 s. Sensorgrams were double referenced using the reference biosensors and a buffer cycle. Steady-state analysis was realized by fitting the αS concentration dependency of the highest response with a simple 1:1 binding model. After normalization, all on and off curves were fitted against simple exponential build-up or decays and led to similar on- and off-rates.

### Solution NMR spectroscopy

Solution NMR experiments were performed on a Bruker Avance III HD^+^ spectrometer operating at 600 MHz ^1^H Larmor frequency, equipped with a triple resonance TCI (^1^H, ^13^C, ^15^N) cryoprobe and shielded z-gradients. If not stated otherwise, all experiments were recorded at 10 °C with an αS concentration of 50 µM in 20 mM sodium phosphate pH 7.4, 50 mM NaCl, 10% (v/v) ^2^H_2_O, and ND concentration was set to 25 µM (one αS per membrane leaflet). All [^1^H-^15^N]-TROSY-HSQC NMR spectra were acquired with 32 scans and 256 indirect increments, processed with TOPSPIN 3.2 (Bruker) and analyzed with CCPN^60^. A full list of measured samples can be found in Supplementary Table 1. For attenuation profiles, peaks were automatically integrated and the ratio of volumes in the presence and absence of NDs plotted against the primary sequence. The raw data without normalization, multi-residue averaging, or manual compensation of peak position is shown. Negative values are results of noise contributions and/or slight peak shifts. Outliers as results of peak overlap and/or ambiguities were removed.

### ThT fluorescence aggregation assays

Three types of aggregation assays were used in this study. Unless otherwise stated, a setup under conditions where αS amyloid fibrils form spontaneously, mainly by interface-driven nucleation and subsequent amplification through fragmentation, was chosen. Experiments under these conditions mainly report on the potential interference of NDs on the lipid-independent aggregation pathways. Here, 50 µM of αS or acetylated αS was mixed with either 25 µM (2:1), 3.125 µM (16:1), or 0.781 µM (64:1) NDs with different lipid compositions. Assays were conducted in 20 mM sodium phosphate buffer pH 7.4 or 20 mM acetate buffer pH 5.3 with 50 mM NaCl, 0.02% NaN_3_, and 10 µM ThT. Unless otherwise stated, triplicates of 120 µl were pipetted into 96-well half-area well plates with non-binding surface (Corning No. 3881, black, clear bottom) containing a glass ball (2.85–3.45 mm diameter) for mixing and incubated at 37 °C for up to 7 days. Orbital shaking at 217 r.p.m. was used for 15 s every 20 min. ThT fluorescence was excited at 445 nm and measured at 485 nm every 20 min with 15 s of shaking before the measurement in a plate reader (Tecan Spark 10 M or Tecan infinite M1000PRO).

*Nucleation-sensitive assays*: Under that minimize the intrinsic nucleation rate (quiescent conditions and protein repellent plate surfaces), lipid bilayers in the form of SUVs can accelerate the nucleation of αS amyloid fibrils^29^. In order to determine whether NDs can have a similarly accelerating effect, we performed ThT aggregation experiments under similar conditions, i.e., where no αS aggregation should be detected in the absence of lipids. These experiments were performed at mildly acidic pH (5.3), as it was recently shown that under these conditions, αS amyloid fibrils can amplify autocatalytically through surface-catalyzed secondary nucleation^49,61^. This should in principle enable even very low primary nucleation rates to be detected through autocatalytic amplification.

*Elongation-sensitive assays*: Seeded experiments using preformed αS fibrils were used to measure the effect of NDs on fibril elongation. Fibril seeds of αS or acetylated αS were prepared as follows: 300 µl of 100 µM αS or acetylated αS was fibrillated at 37 °C and 800 r.p.m. for 3 days in a 2 ml tube containing a glass ball in a Thermomixer (Eppendorf). The fibril solution was diluted to 50 µM and sonicated with a tip sonicator (Bandelin Sonopuls HD3200, BANDELIN electronic) at 10% power (20 W) for 60 s, with 1 s pulses on and 4 s off in between. Seed solution was diluted 20-fold for the aggregation assays (2.5 µM, 5%).

*Assay analysis*: Kinetic curves were corrected by subtracting the curve of buffer (containing NDs) in the presence of ThT and normalized to the highest fluorescence intensity (in line with comparable fibril mass seen in SDS-polyacrylamide gel electrophoresis after the aggregation assay). The corresponding triplicates are shown as transparent circles in order to visualize the reproducibility of each experiment. Data fits were obtained using a simple sigmoidal function and the Abscissa 2D plot tool (by Rüdiger Brühl). In the case of quiescent nucleation and seeded assays, no normalization was applied and data were recorded without the presence of glass balls and without plate shaking.

### SDS–polyacrylamide gel electrophoresis

In order to compare the amounts of soluble and fibrillated αS or acetylated αS in the aggregation samples, 100 µl of each triplicate sample were taken out of the well plate, combined in 1.5 ml tubes, and spun down at 20,000 × *g* and 20 °C for 30 min. Supernatants (~ 290 µl) were removed and pellets were resuspended in 280 µl buffer, and SDS-sample buffer (4-fold) was added. Samples were boiled for 15 min at 98 °C and subsequently 10 µl were loaded onto a 15% SDS-gel together with standards of αS or acetylated αS and NDs.

### DNP NMR spectroscopy

Magic-angle spinning solid-state DNP experiments were performed on a Bruker Avance III HD spectrometer operating at 600 MHz, equipped with a 395.18 GHz second-harmonic gyrotron and a 3.2 mm ^1^H,^13^C,^15^N triple resonance low-temperature magic-angle-spinning probe. Data were collected at 100 K, 9 kHz magic angle spinning speed, and 9 W continuous-wave microwave power. The samples were prepared from sparsely labeled non-acetylated αS (250 µg) in the presence or in the absence of 2:1 molar ratio of 100% POPG NDs, and filled into 3.2 mm sapphire rotors. Final buffer conditions in the sample were 15 mM sodium phosphate pH 7.4, 25 mM sodium chloride, 30% ^2^H_2_O, 60% glycerol-d_6_, and 2.5 mM AMUPOL^62^. Two-dimensional [^13^C-^13^C]-proton-driven spin diffusion experiments with 1 s mixing time were performed. It is noteworthy  that this DNP setup also allows a more detailed quantatitive analysis of different αS conformational ensembles and the effects of different polarization transfers as well^63^. ^1^H decoupling using SPINAL64 with a decoupling field of 104 kHz was employed during evolution and detection periods. Both experiments were conducted using 300 *t*_1_ increments with 16 and 48 scans each for αS in the absence and in the presence of NDs, respectively. A recycle delay of 5 s was used in both experiments. Both spectra were processed using Topspin 3.2 (Bruker) using identical parameters with squared and shifted sine bell function (qsine 2.5) for apodization.

### MD simulations

As starting conformation for the MD simulations, the NMR structure of micelle-bound αS (PDB 1XQ8) was used, considering only the first 61 residues in order to concentrate on the membrane-binding region of αS.

The Amber99sb-ILDN force field^64^ was used for αS, which was simulated in its non-acetylated form (i.e., with NH3 + at the N-terminus) and with a C-terminal N-methyl amide capping group to account for the fact that αS would continue beyond residue 61. All lysine side chains were modeled as positively charged, glutamate and aspartate as negatively charged, whereas glutamine and histidine residues were considered to be neutral corresponding to pH 7.4. The protein was placed either 0.5 nm or 1.5 nm above the membrane surface. A starting orientation with the negatively charged side chains pointing away from the membrane and the lysine side chains being oriented toward the membrane surface were chosen (Fig. 4b). For modeling the lipid bilayer, membrane patches consisting of POPC/POPG (1:1) or DMPC/DMPG (1:1) involving 512 lipids (256 lipids per leaflet) were built using CHARMM-GUI^65^ and modeled with Slipids force field parameters^66,67^. Before αS was added, both lipid bilayers were solvated and simulated for 500 ns (POPC/POPG) or 1000 ns (DMPC/DMPG) to obtain relaxed membranes. Here, the same simulation procedure was employed as described below. αS was placed above the membrane, the protein–membrane complex solvated using the TIP3 water model, and Na^+^ and Cl^–^ were added to neutralize the system and to mimic the Na + concentration used in the experiments. The ion parameters of Smith and Dang^68^ were used. The system was then subjected to steepest descent energy minimization, followed by MD equilibration in the constant number (N), volume (V) and temperature (T) (NVT) ensemble for 1 ns at 10 °C using the V-rescale thermostat^69^ with a time constant of 0.5 ps and separate temperature coupling for the protein, membrane, and water/ions. Afterwards, 1 ns of constant number (N), pressure (P) and temperature (T) (NPT) equilibration was performed using the Nose–Hoover thermostat^70,71^ and Parrinello–Rahman barostat^72^ with semi-isotropic pressure scaling, a reference pressure of 1 bar, a time constant of 10.0 ps, and an isothermal compressibility of 4.5 × 10^–5^ bar^–1^. During both equilibration steps, restraints were applied to the positions of the P-atoms of the lipids and terminal C-atoms of their tails with a force constant of 1000 kJ mol^–1^ nm^−2^. All bond lengths were constrained using the Lincs algorithm^73^. The Coulombic interactions were calculated using the Particle mesh Ewald method^74,75^ with a cutoff value of 1.0 nm for the short-range interactions and a Fourier spacing of 0.12 nm. The cutoff value for the van der Waals interactions was set at 1.4 nm. Periodic boundary conditions were employed in all directions. For the MD production runs, the same settings as for the NPT equilibration were used, except that all position restraints were removed. All MD simulations were performed at 10 °C with a time step of 2 fs for integration using the GROMACS 4.6 MD package^76^. For the analysis, which was performed using Gromacs and Membrainy tools^77^, only the last 250 ns of each production run was used.

A full list of simulations can be found in Supplementary Table 2.

### Size exclusion chromatography

For analytical SEC, an Akta pure systems equipped with a Superdex 200 13/300 gl column (GE Healthcare) was used. After equilibration with two column volumes of running buffer (20 mM sodium phosphate pH 7.4, 50 mM NaCl), samples of approximately 5 µM NDs, 10 µM αS, or a mix thereof were injected at a flowrate of 0.5 ml min^–1^. In order to assay stability of the NDs in the presence of αS, the same mix was incubated 24 h at room temperature and injected afterwards.

### Differential scanning calorimetry

Samples of approximately 5 µM NDs of different types (and if stated 10 µM αS) in running buffer (20 mM sodium phosphate pH 7.4, 50 mM NaCl) were degassed for at least 20 min at 30 °C and measured in a Microcal VP-DSC instrument (Malvern Instruments). After equilibration and a pre-scan delay of 20 min, the thermograms were acquired up-scan from 5 °C to 45 °C at a scanning rate of 0.5 °C min^–1^ and corrected by subtracting the thermogram of buffer and adjusting the baseline to zero at 45 °C (*y-*axis molarity refers to estimated amounts of lipids).

### Atomic force microscopy

AFM images were taken in air, using a Nanowizard III atomic force microscope (JPK, Berlin). Samples were taken at the end of aggregation experiments and diluted in pure water to approximately 1 µM protein concentration. Ten microliters of sample were added onto freshly cleaved mica and left to dry. Then, they were gently rinsed with water to remove excess salt. Imaging was performed using tapping mode with NSC 36 cantilevers (MikroMasch), with resonant frequencies between 70 and 150 kHz.

### Data availability

The datasets generated during and/or analysed during the current study are available from the corresponding author on request. NMR chemical shift asignments are in line with data already deposited in the Biological Magnetic Resonance Data Bank, e.g., under accession codes 19350 (acetlyated) and 19337 (non-acetylated)^78^.

## Electronic supplementary material

Supplementary Information

## References

[1] Spillantini MG (1997). Alpha-synuclein in lewy bodies. Nature.

[2] Luk KC (2012). Pathological alpha-synuclein transmission initiates parkinson-like neurodegeneration in nontransgenic mice. Science (New York, N. Y.).

[3] Jucker M, Walker LC (2013). Self-propagation of pathogenic protein aggregates in neurodegenerative diseases. Nature.

[4] Tuttle MD (2016). Solid-state NMR structure of a pathogenic fibril of full-length human alpha-synuclein. Nat. Struct. Mol. Biol..

[5] Gitler AD (2008). The Parkinson’s disease protein alpha-synuclein disrupts cellular Rab homeostasis. Proc. Natl Acad. Sci. USA.

[6] Bellani S (2010). The regulation of synaptic function by alpha-synuclein. Commun. Integr. Biol..

[7] Diao J (2013). Native alpha-synuclein induces clustering of synaptic-vesicle mimics via binding to phospholipids and synaptobrevin-2/VAMP2. eLife.

[8] Fusco G (2016). Structural basis of synaptic vesicle assembly promoted by alpha-synuclein. Nat. Commun..

[9] Zhu M, Li J, Fink AL (2003). The association of alpha-synuclein with membranes affects bilayer structure, stability, and fibril formation. J. Biol. Chem..

[10] Dikiy I, Eliezer D (2012). Folding and misfolding of alpha-synuclein on membranes. Biochim. Et. Biophys. Acta.

[11] Butterfield SM, Lashuel HA (2010). Amyloidogenic protein-membrane interactions: Mechanistic insight from model systems. Angew. Chem. Int. Ed. Engl..

[12] Auluck PK, Caraveo G, Lindquist S (2010). Alpha-synuclein: membrane interactions and toxicity in parkinson’s disease. Annu. Rev. Cell Dev. Biol..

[13] Nemani VM (2010). Increased expression of alpha-synuclein reduces neurotransmitter release by inhibiting synaptic vesicle reclustering after endocytosis. Neuron.

[14] Dikiy I, Eliezer D (2014). N-terminal acetylation stabilizes n-terminal helicity in lipid- and micelle-bound alpha-synuclein and increases its affinity for physiological membranes. J. Biol. Chem..

[15] Rhoades E, Ramlall TF, Webb WW, Eliezer D (2006). Quantification of α-synuclein binding to lipid vesicles using fluorescence correlation spectroscopy. Biophys. J..

[16] Jo E, McLaurin J, Yip CM, St George-Hyslop P, Fraser PE (2000). Alpha-synuclein membrane interactions and lipid specificity. J. Biol. Chem..

[17] Bodner CR, Dobson CM, Bax A (2009). Multiple tight phospholipid-binding modes of alpha-synuclein revealed by solution NMR spectroscopy. J. Mol. Biol..

[18] Theillet FX (2016). Structural disorder of monomeric alpha-synuclein persists in mammalian cells. Nature.

[19] Bodner CR, Maltsev AS, Dobson CM, Bax A (2010). Differential phospholipid binding of alpha-synuclein variants implicated in parkinson’s disease revealed by solution NMR spectroscopy. Biochemistry.

[20] Ulmer TS, Bax A, Cole NB, Nussbaum RL (2005). Structure and dynamics of micelle-bound human alpha-synuclein. J. Biol. Chem..

[21] Jao CC, Hegde BG, Chen J, Haworth IS, Langen R (2008). Structure of membrane-bound alpha-synuclein from site-directed spin labeling and computational refinement. Proc. Natl Acad. Sci. USA.

[22] Drescher M (2008). Antiparallel arrangement of the helices of vesicle-bound alpha-synuclein. J. Am. Chem. Soc..

[23] Fusco G (2014). Direct observation of the three regions in alpha-synuclein that determine its membrane-bound behaviour. Nat. Commun..

[24] Eliezer D, Kutluay E, Bussell R, Browne G (2001). Conformational properties of alpha-synuclein in its free and lipid-associated states. J. Mol. Biol..

[25] Georgieva ER, Ramlall TF, Borbat PP, Freed JH, Eliezer D (2008). Membrane-bound alpha-synuclein forms an extended helix: long-distance pulsed esr measurements using vesicles, bicelles, and rodlike micelles. J. Am. Chem. Soc..

[26] Drescher M (2008). Spin-label epr on alpha-synuclein reveals differences in the membrane binding affinity of the two antiparallel helices. Chembiochem.

[27] Zhu M, Fink AL (2003). Lipid binding inhibits alpha-synuclein fibril formation. J. Biol. Chem..

[28] Zhao H, Tuominen EK, Kinnunen PK (2004). Formation of amyloid fibers triggered by phosphatidylserine-containing membranes. Biochemistry.

[29] Galvagnion C (2015). Lipid vesicles trigger alpha-synuclein aggregation by stimulating primary nucleation. Nat. Chem. Biol..

[30] Galvagnion C (2016). Chemical properties of lipids strongly affect the kinetics of the membrane-induced aggregation of alpha-synuclein. Proc. Natl Acad. Sci. USA.

[31] Bayburt TH, Grinkova YV, Sligar SG (2002). Self-assembly of discoidal phospholipid bilayer nanoparticles with membrane scaffold proteins. Nano Lett..

[32] Zhang Z (2014). Ca(2+) modulating alpha-synuclein membrane transient interactions revealed by solution NMR spectroscopy. Biochim. Et. Biophys. Acta.

[33] Thomaier M (2016). High-affinity binding of monomeric but not oligomeric amyloid-beta to ganglioside GM1 containing nanodiscs. Biochemistry.

[34] Viegas A, Viennet T, Etzkorn M (2016). The power, pitfalls and potential of the nanodisc system for NMR-based studies. Biol. Chem..

[35] Denisov IG, Grinkova YV, Lazarides AA, Sligar SG (2004). Directed self-assembly of monodisperse phospholipid bilayer nanodiscs with controlled size. J. Am. Chem. Soc..

[36] Her C (2016). The charge properties of phospholipid nanodiscs. Biophys. J..

[37] Inagaki S (2012). Modulation of the interaction between neurotensin receptor NTS1 and Gq protein by lipid. J. Mol. Biol..

[38] Ouberai MM (2013). Alpha-synuclein senses lipid packing defects and induces lateral expansion of lipids leading to membrane remodeling. J. Biol. Chem..

[39] Eichmann C (2016). Preparation and characterization of stable alpha-synuclein lipoprotein particles. J. Biol. Chem..

[40] Comellas G, Lemkau LR, Zhou DH, George JM, Rienstra CM (2012). Structural intermediates during α-synuclein fibrillogenesis on phospholipid vesicles. J. Am. Chem. Soc..

[41] Hagn F, Etzkorn M, Raschle T, Wagner G (2013). Optimized phospholipid bilayer nanodiscs facilitate high-resolution structure determination of membrane proteins. J. Am. Chem. Soc..

[42] Etzkorn M (2013). Cell-free expressed bacteriorhodopsin in different soluble membrane mimetics: biophysical properties and NMR accessibility. Structure.

[43] Iyer A (2016). The impact of N-terminal acetylation of alpha-synuclein on phospholipid membrane binding and fibril structure. J. Biol. Chem..

[44] Pirc K, Ulrih NP (2015). Αlpha-synuclein interactions with phospholipid model membranes: Key roles for electrostatic interactions and lipid-bilayer structure. Biochim. Biophys. Acta.

[45] Lokappa SB (2014). Sequence and membrane determinants of the random coil-helix transition of alpha-synuclein. J. Mol. Biol..

[46] Rabe M (2013). On-surface aggregation of α-synuclein at nanomolar concentrations results in two distinct growth mechanisms. ACS Chem. Neurosci..

[47] Vacha R, Linse S, Lund M (2014). Surface effects on aggregation kinetics of amyloidogenic peptides. J. Am. Chem. Soc..

[48] Campioni S (2014). The presence of an air-water interface affects formation and elongation of alpha-synuclein fibrils. J. Am. Chem. Soc..

[49] Gasper R (2017). Secondary nucleation of monomers on fibril surface dominates α-synuclein aggregation and provides autocatalytic amyloid amplification. Quart. Rev. Biophys..

[50] Siemer AB, Huang KY, McDermott AE (2012). Protein linewidth and solvent dynamics in frozen solution nmr. PLoS ONE.

[51] Hong M, Jakes K (1999). Selective and extensive 13C labeling of a membrane protein for solid-state NMR investigations. J. Biomol. NMR.

[52] Davidson WS, Jonas A, Clayton DF, George JM (1998). Stabilization of alpha-synuclein secondary structure upon binding to synthetic membranes. J. Biol. Chem..

[53] Iyer A, Petersen NO, Claessens MM, Subramaniam V (2014). Amyloids of alpha-synuclein affect the structure and dynamics of supported lipid bilayers. Biophys. J..

[54] Reynolds NP (2011). Mechanism of membrane interaction and disruption by alpha-synuclein. J. Am. Chem. Soc..

[55] Burre J (2010). Alpha-synuclein promotes snare-complex assembly in vivo and in vitro. Sci. (New York, N. Y.).

[56] Perni M (2017). A natural product inhibits the initiation of alpha-synuclein aggregation and suppresses its toxicity. Proc. Natl Acad. Sci. USA.

[57] Johnson M, Coulton AT, Geeves MA, Mulvihill DP (2010). Targeted amino-terminal acetylation of recombinant proteins in *E. coli*. PLoS ONE.

[58] Hoyer W (2002). Dependence of α-synuclein aggregate morphology on solution conditions. J. Mol. Biol..

[59] Bayburt TH, Carlson JW, Sligar SG (1998). Reconstitution and imaging of a membrane protein in a nanometer-size phospholipid bilayer. J. Struct. Biol..

[60] Vranken WF (2005). The CCPN data model for NMR spectroscopy: development of a software pipeline. Proteins.

[61] Buell AK (2014). Solution conditions determine the relative importance of nucleation and growth processes in alpha-synuclein aggregation. Proc. Natl Acad. Sci. USA.

[62] Sauvee C (2013). Highly efficient, water-soluble polarizing agents for dynamic nuclear polarization at high frequency. Angew. Chem. Int. Ed. Engl..

[63] Uluca B (2018). DNP-enhanced MAS NMR: A tool to snapshot conformational ensembles of alpha-synuclein in different states. Biophys. J.

[64] Lindorff-Larsen K (2010). Improved side-chain torsion potentials for the amber ff99sb protein force field. Proteins.

[65] Lee J (2016). Charmm-GUI input generator for namd, gromacs, amber, openmm, and charmm/openmm simulations using the charmm36 additive force field. J. Chem. Theory Comput..

[66] Jambeck JP, Lyubartsev AP (2012). Derivation and systematic validation of a refined all-atom force field for phosphatidylcholine lipids. J. Phys. Chem. B.

[67] Jambeck JP, Lyubartsev AP (2013). Another piece of the membrane puzzle: extending slipids further. J. Chem. Theory Comput..

[68] Smith DE, Dang LX (1994). Computer simulations of nacl association in polarizable water. J. Chem. Phys..

[69] Bussi G, Donadio D, Parrinello M (2007). Canonical sampling through velocity rescaling. J. Chem. Phys..

[70] Nosé S (1984). A molecular dynamics method for simulations in the canonical ensemble. Mol. Phys..

[71] Hoover WG (1985). Canonical dynamics: equilibrium phase-space distributions. Phys. Rev. A: General. Phys..

[72] Parrinello M, Rahman A (1981). Polymorphic transitions in single crystals: a new molecular dynamics method. J. Appl. Phys..

[73] Hess B, Bekker H, Berendsen HJC, Fraaije JGEM (1997). Lincs: a linear constraint solver for molecular simulations. J. Comput. Chem..

[74] Darden T, York D, Pedersen L (1993). Particle mesh ewald: an n⋅log(n) method for ewald sums in large systems. J. Chem. Phys..

[75] Essmann U (1995). A smooth particle mesh ewald method. J. Chem. Phys..

[76] Hess B, Kutzner C, van der Spoel D, Lindahl E (2008). Gromacs 4: algorithms for highly efficient, load-balanced, and scalable molecular simulation. J. Chem. Theory Comput..

[77] Carr M, MacPhee CE (2015). Membrainy: a ‘smart’, unified membrane analysis tool. Source Code Biol. Med..

[78] Kang L, Janowska MK, Moriarty GM, Baum J (2013). Mechanistic insight into the relationship between N-terminal acetylation of α-synuclein and fibril formation rates by NMR and fluorescence. PLoS ONE.

