# Supplementary material for: Structural insights from lipid-bilayer nanodiscs link α-Synuclein membrane-binding modes to amyloid fibril formation
Source: Commun Biol. 2018 May 3;1:44. doi: 10.1038/s42003-018-0049-z (PMC6123806; doi:10.1038/s42003-018-0049-z)
Supplement: Supplementary file 1 — Supplementary Information [file 42003_2018_49_MOESM1_ESM.pdf]

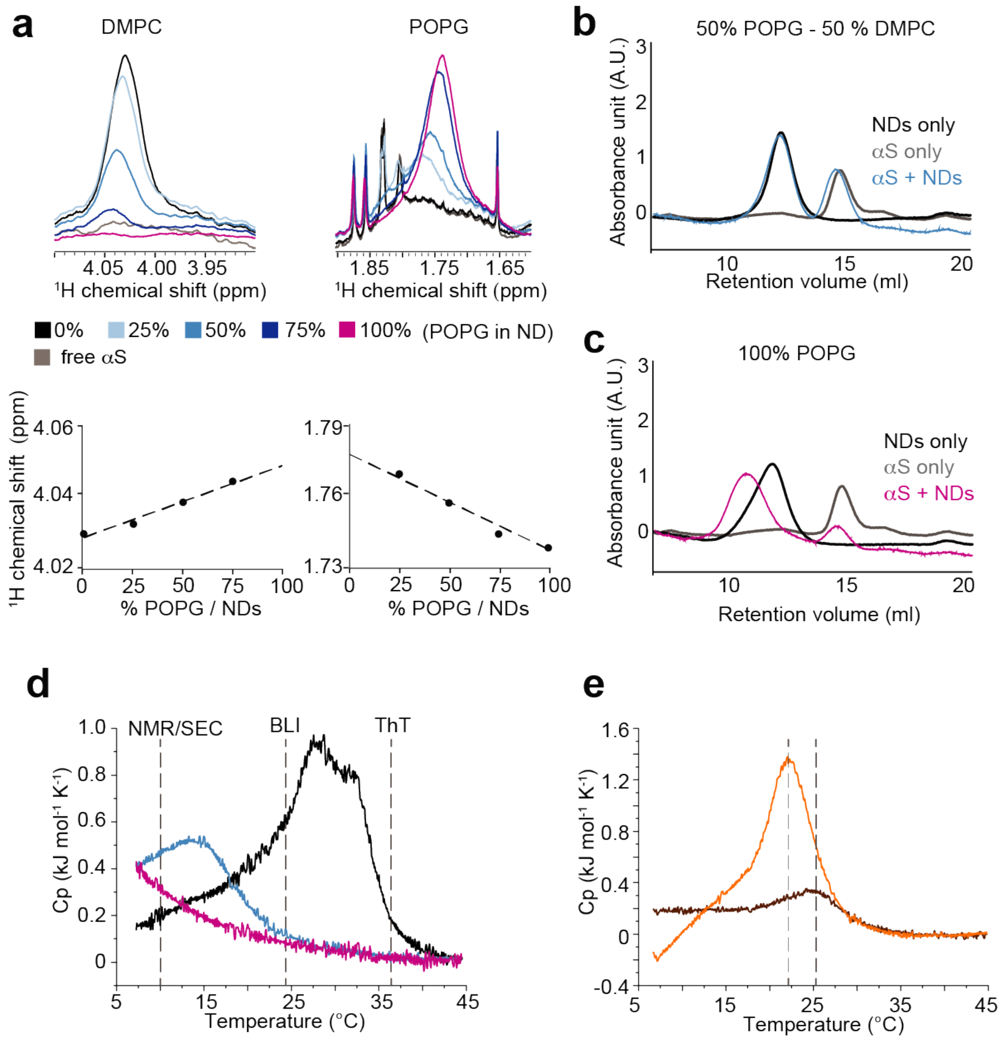

Supplementary Figure 1. Formation of lipid mixtures in NDs, lipid phase transitions and stability of αS association. (**a**) NMR ^{1}H-1D spectra of αS in the presence of NDs containing different POPG-to-DMPC ratios corresponding to the samples shown in Fig. 1a-b. Signals from either DMPC (left, choline methyl groups) or POPG (right, ^{1}H next to the unsaturation) are displayed. The volumes of the peaks reflect within an error of approximately 10% the aimed lipid compositions. In addition to volume changes also chemical shifts perturbations for both lipid specific peaks are visible and follow a rather linear dependence on the composition. The NMR data therefore report on both the presence and mixing of both lipid types in single nanodiscs. (**b**) Size exclusion chromatography (SEC) profiles (Superdex 200 13/300 gl, GE Healthcare) of αS alone (grey), 50% POPG NDs alone (black) and both after mixing (same amounts as in the isolated case) and incubation for 24h at room temperature (blue). The clear separation of αS from NDs in the mixture points to a fast-kinetic exchange between free and bound αS. The conserved total absorbance additionally confirms the stability of the NDs in the presence of αS. (**c**) Same as in (b) but using 100% POPG NDs. The SEC profile of the mixture of αS with 100% POPG NDs shows a clear reduction of free αS and a size increase of the ND peak, pointing to a strong interaction between the two components. (**d**) Differential scanning calorimetry (DSC) thermograms of nanodiscs prepared with either 100% DMPC (black), 100% POPG (purple) or a 50%-50% mixture thereof (blue), showing phase transitions temperatures around 28°C, below 5°C and 13°C, respectively. Temperatures at which different measurements were performed are highlighted. (**e**) DSC profile of 100% DMPG NDs in the absence (brown) or presence of 2 molar equivalent αS (orange). While the presence of αS leads to a lower phase transition temperatures of the lipid bilayer, in line with what was reported using SUVs[1], the transition temperature differences is much smaller for NDs (ΔT_m=2.5 °C) as compared to SUVs (ΔT_m=11 °C) in line with an increase stability of the NDs. Note that a more detailed discussion of the stability of the nanodiscs is given in the Supplementary Note 1 (*vide infra*).

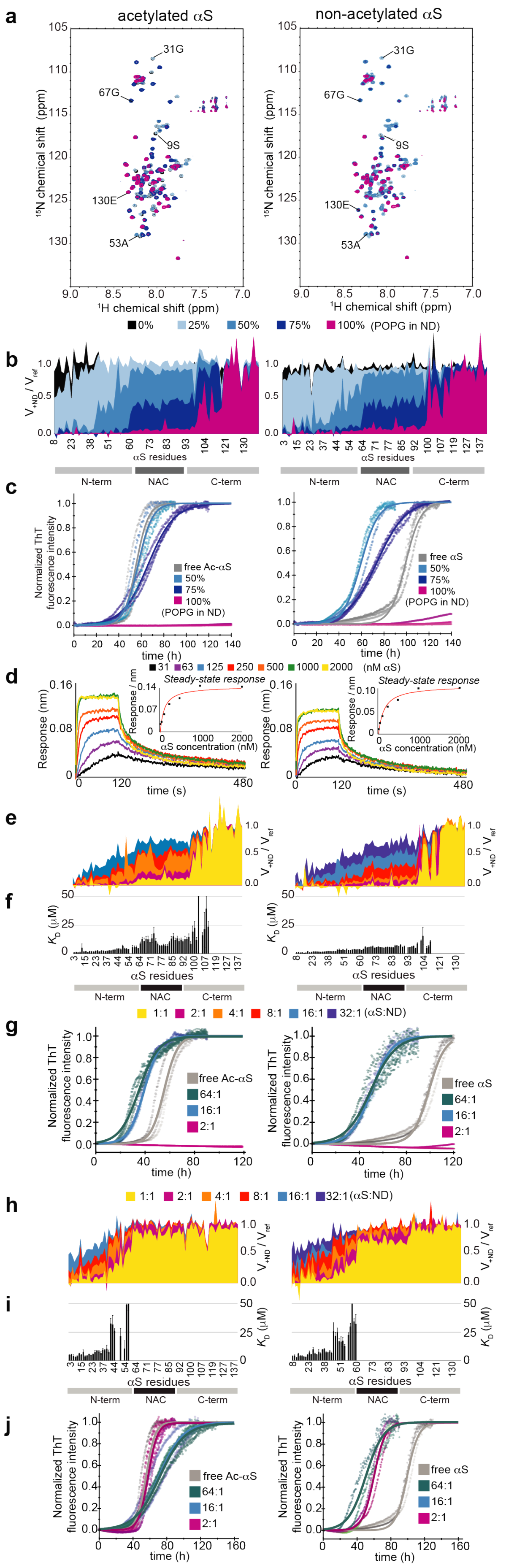

Supplementary Figure 2. N-terminal acetylation has moderate effect on membrane association and leads to different behavior in aggregation assays. Comparison of data recorded on acetylated αS (left hand side) and non-acetylation αS (right hand side). Note that data of acetylated αS represents the data shown in the main manuscript and is just added to facilitate a direct comparison. (**a**, *analog Fig. 1a*) $[^{15}N$-$^{1}H]$-TROSY-HSQC NMR spectra in the absence (grey) or in the presence of 25 µM NDs containing an increasing amount of anionic lipids. **(b**, *analog Fig. 1b,c***)** Corresponding NMR attenuation profiles, i.e. the ratio of peak volumes in the presence and absence of NDs, are plotted against αS primary sequence. In line with previous findings[9], N-terminal acetylation leads to clear chemical shift perturbations in the NMR spectra for the first 10 residues of αS. The peaks which are already shifted in free αS due to the acetylation are also the ones that are affected most by the presence of nanodiscs with low amount of charges (close to physiological concentration). Data show rather small but significant increase in the membrane association of the first 15 residues due to the N-terminal acetylation, which is in line with previous observation using SUVs[10,11]. αS acetylation is known to increase N-terminal helix propensity[9,12], which may facilitate formation of the initial binding mode and be of significance for naturally occurring processes.

(**c**, *analog Fig. 1e*) αS aggregation assays (normalized ThT fluorescence) in the absence and presence of NDs with indicated POPG content. While aggregation behavior in the presence of NDs is in general very similar for acetylated and non-acetylated αS in all tested conditions, it is very different in the absence of NDs. In the absence of NDs the ThT kinetic data of non-acetylated αS reproducibly have a strongly delayed reference kinetic curve under the applied conditions as compared to the acetylated reference. In the setup used, primary nucleation processes are likely to happen at the air-water or plate-water interface[13,14], thus a lower hydrophobic propensity of non-acetylated αS could explain this effect. While this may be the dominant process in the absence of lipids, it may not be the case anymore in the presence of NDs [1], either because NDs shield these interfaces or because nucleation happens primarily at the membrane surface. The much lower differences due to acetylation state in the presence of NDs fit this explanation, as well as additional tests we ran using different types of plates (data not shown). Higher order processes, namely different fragmentation behaviors, can however not be excluded.

(**d**, *analog Fig. 3a*) BLI data with immobilized 100% POPG NDs. BLI kinetics show a (slightly) higher membrane affinity of the acetylated (K_D of 67 ± 17 nM) than the non-acetylated αS (K_D of 95 ± 14 nM).

(**e+f**, *analog Fig. 3b+c*) NMR titration data with 100% POPG NDs show a slightly increased membrane affinity of the NAC region for the non-acetylated αS. At current stage, it is not easy to explain why a modification at the N-terminus will affect the lipid interaction of a protein region that is sequentially separated by roughly 60 residues. Such a behavior could however either be related to intermolecular interactions and/or long range intramolecular interactions (in a 'horseshoe'-conformation) that may or may not be artificially introduced by the limited surface area of the NDs.

(**g**, *analog Fig. 3d*) Normalized ThT fluorescence kinetic curves for selected αS-to-ND ratios using 100% POPG NDs.

(**h+i**, *analog Fig. 3f+g*) NMR titration data with 50% POPG NDs show similar affinities of N-terminal residues for acetylated and non-acetylated αS.

(**j**, *analog Fig. 3h*) Normalized ThT fluorescence kinetic curves for selected αS-to-ND ratios using 50% POPG NDs.

Overall, it appears that the biggest effect of acetylation on aggregation is related to assay parameters that are normally not the matter of interest, which nevertheless may be important for future studies[15]. Still the results from systematic measurement of the effects of N-terminal acetylation via different methods point to subtle changes in membrane interaction in respect to NAC region specific affinities at high lipid charge densities as well as to N-terminal binding at a lipid charge density comparable with the overall composition found for membranes in e.g. synaptic vesicles[16].

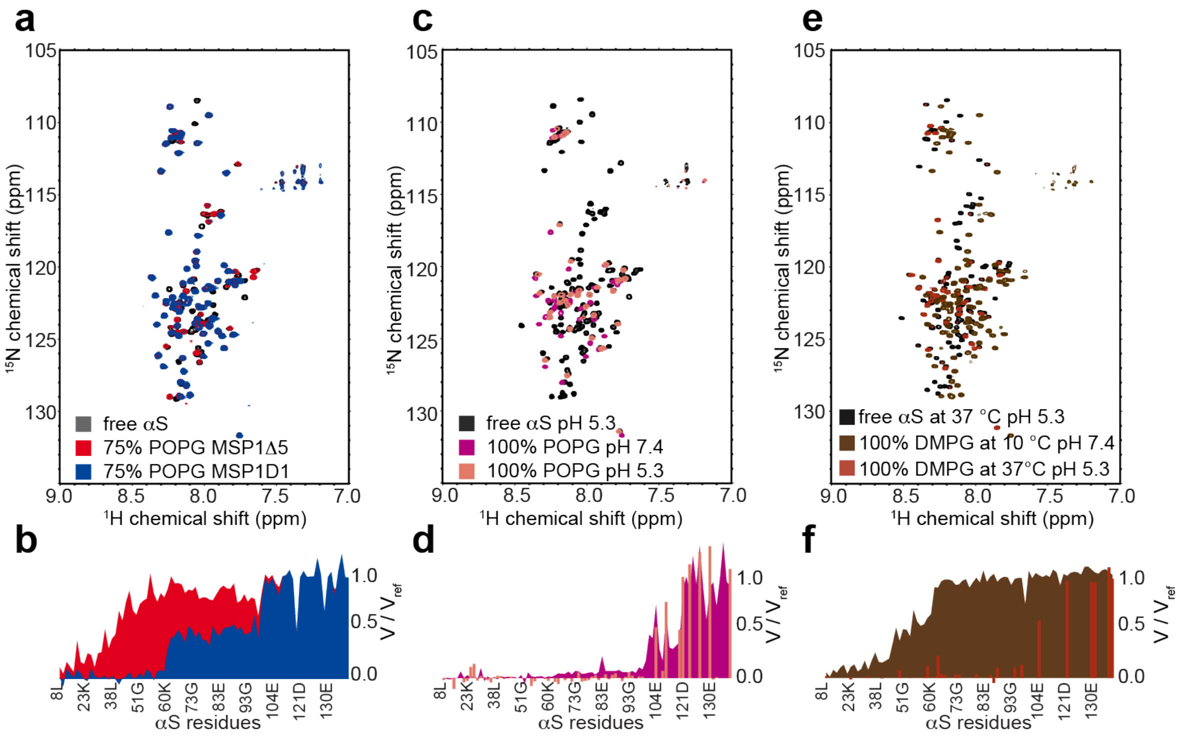

Supplementary Figure 3. αS-ND interactions using different MSP constructs, lower pH and higher temperature. NMR spectra (**a**) and the respective attenuation profiles (**b**) of [^{15}N]-acetylated-αS (50 µM) in the absence or in the presence of 25 µM NDs with 75% POPG content, assembled using the regular MSP1D1 construct (blue) or with the smaller MSP1Δ5 (red). Spectra (**c**) and attenuation profiles (**d**) corresponding to the binding of αS to NDs containing 100% POPG lipids at pH 7.4 (purple) and pH 5.3 (pale orange). Transferable assignments (bars) show that no significant difference in binding mode is visible upon pH variation. Spectra (**e**) and attenuation profiles (**f**) corresponding to the binding of αS to NDs containing 100% DMPG lipids in their gel phase (10°C, brown) or their fluid phase (37°C, red). Since normally measurement at 37 °C leads to considerable peak loss of N-terminal αS residues due to water exchange processes, the spectrum was recorded at pH 5.3 (counteracting water exchange). Note that the pH shift alone has no significant effect on binding (see (**c**) and (**d**)). Reference spectra of respective free αS are shown in dark grey. Note that a more detailed discussion about the NMR detection of the membrane-bound state is given in the Supplementary Note 2 (*vide infra*).

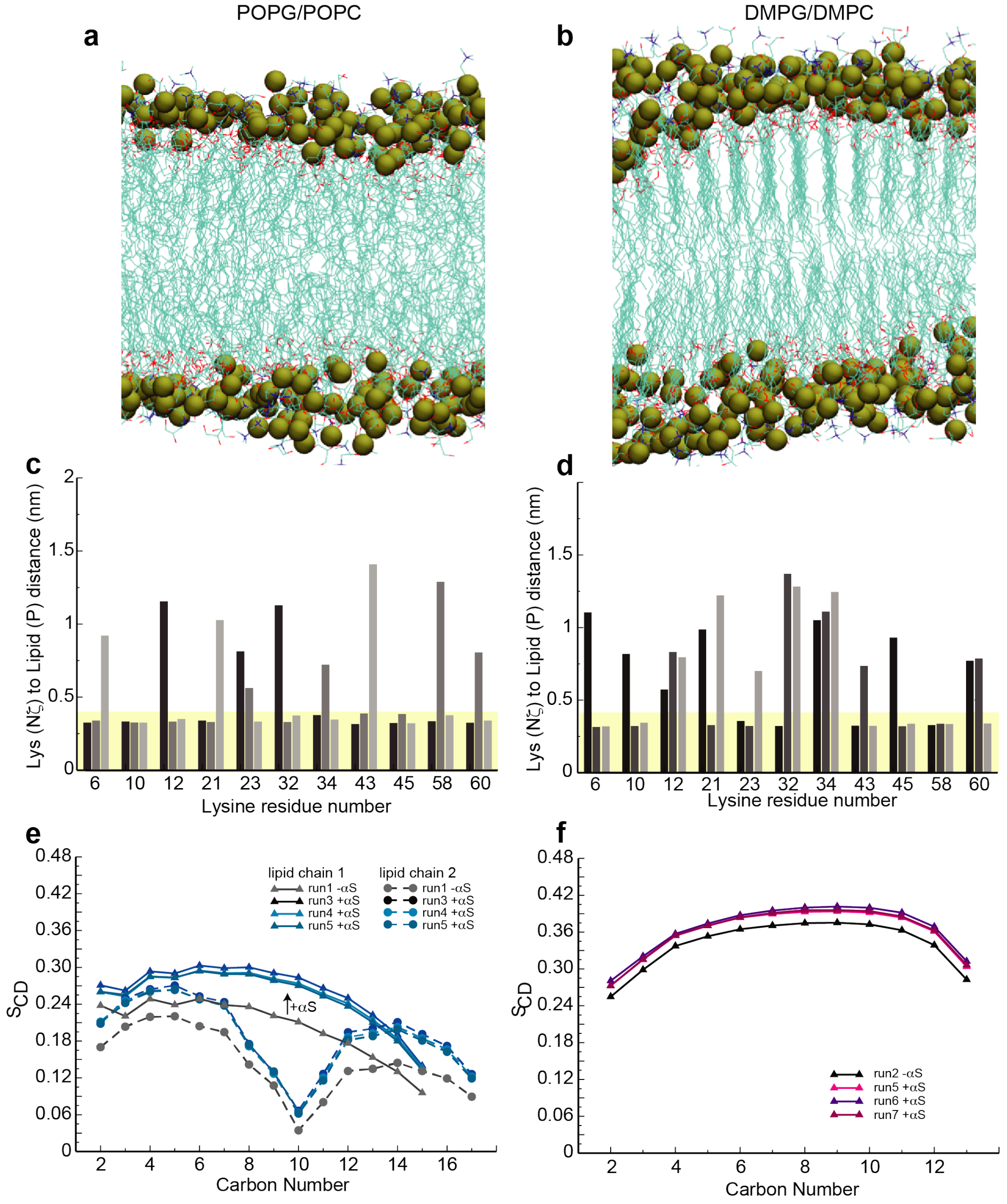

Supplementary Figure 4. Lipid properties and αS-membrane interactions as seen by MD simulations. (**a,b**) Molecular arrangement of lipid bilayer in the absence of αS for POPG/POPC lipids in fluid phase (**a**) and DMPG/DMPC lipids in gel phase (**b**) (end of run1/run2, Table S2). (**c,d**) Distances for indicated lysine residues from Lys-Nξ to the phosphorus atom of the next anionic lipid as found in different runs of MD simulations for POPG/POPC (c, individual bars for run3-5, Table S2) and DMPG/DMPC (d, individual bars for run5-7, Table S2) membranes. Distances that could promote lipid-mediated salt bridges are highlighted in yellow. (**e,f**) Calculated order parameters for indicated carbon atoms of the lipid fatty acids with and without αS for POPG/POPC (**e**) and DMPG/DMPC (**f**) membranes.

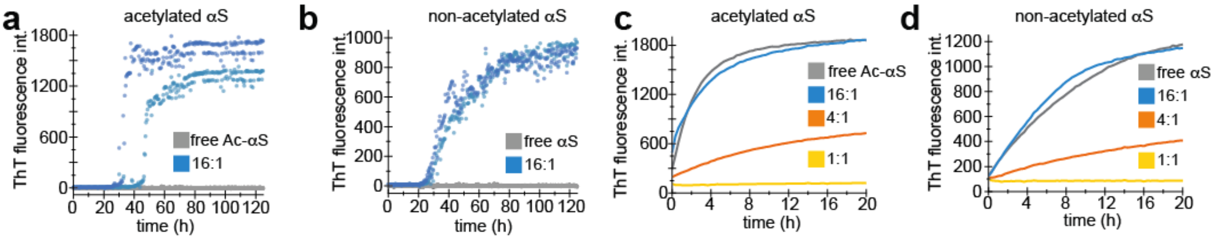

Supplementary Figure 5. Amyloid fibril nucleation and elongation in the presence of 100% POPG ND. (**a,b**) Quiescent nucleation assay of acetylated αS (**a**) and non-acetylated αS (**b**) in the absence (grey, duplicates) and presence of 100% POPG NDs (duplicates in light and dark blue, note that conditions shown in (a) are identical to those shown in Fig. 3e). (**c,d**,) Raw data of quiescent ThT fluorescence aggregation assay in the presence of 2.5% preformed seeds for acetylated (c) or non-acetylated (d) αS for different molar ratios (see color code) of 100% POPG NDs. While at high molar excess of αS (16:1, αS-to-ND), according to our NMR data, nearly all αS monomers should interact through their most N-terminal regions with the NDs, no significant effect on the elongation rate was observed (c,d, blue). At molar ratios of 4:1 (orange) a decrease in elongation is observed, however at this ratio our NMR data clearly indicate that all αS monomers are interacting with the NDs. Nevertheless, in both cases (molar ratios of 16:1 and 4:1) larger fractions of the monomer population should still have accessible NAC regions, which may explain their ability to participate in the fibril elongation process. However, the limited interaction surface may also influence the dynamic nature of membrane association leading to a certain population of free monomeric αS at any given time.

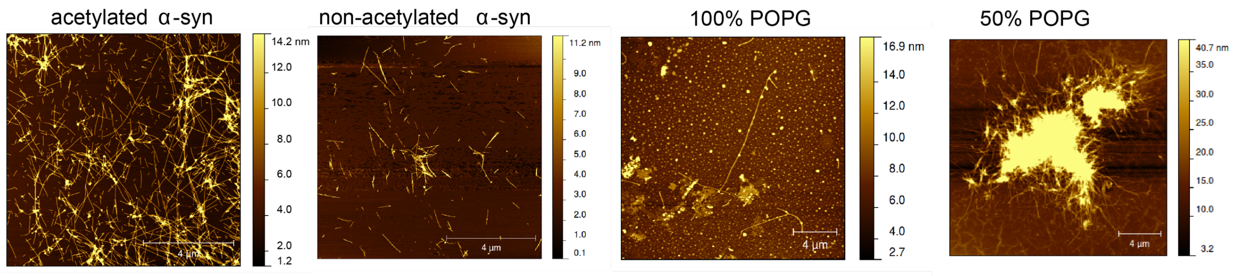

Supplementary Figure 6. αS fibrils grown in the absence and presence of NDs have similar morphologies. AFM images of αS amyloid fibrils formed using acetylated αS and non-acetylated αS as well as acetylated αS in the presence of NDs with indicated lipid composition (from left to right). While no in depth AFM characterization is attempted here, our data indicates that the morphology of all tested samples is rather similar (mostly mature fibrils), in contrast to previous results reported for aggregation in the presence of liposomes[1] (thin and curly fibrils). This observation suggests that the interplay between the lipids and the αS and potential incorporation of lipids into fibrils is different in the case of nanodiscs (in line with a more stable environment compared to liposomes).

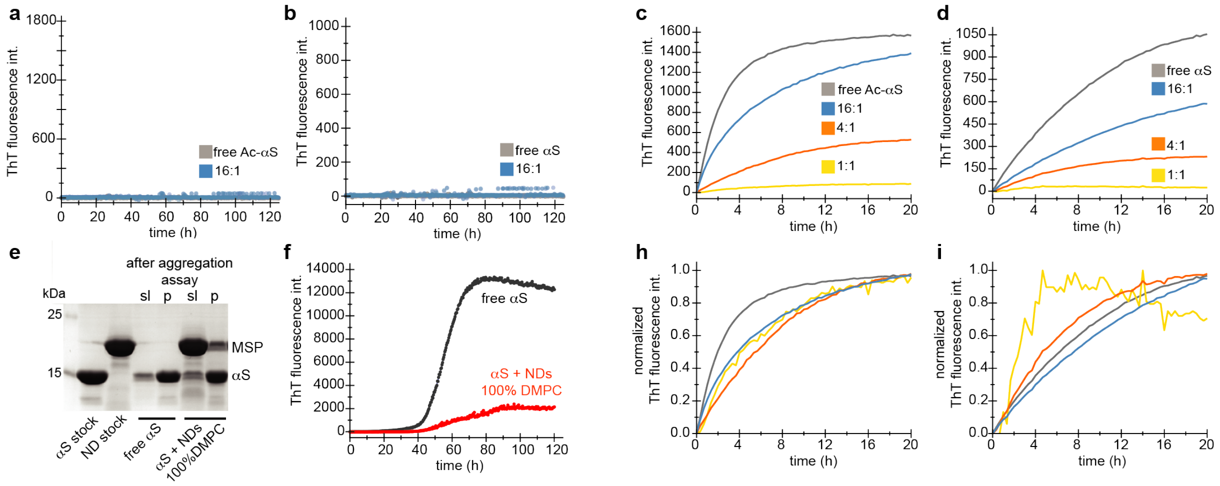

Supplementary Figure 7. Amyloid fibril nucleation and elongation in the presence of 50% POPG ND and rationale for normalization of ThT data. (**a,b**) Quiescent nucleation assay of acetylated αS (**a**) and non-acetylated (**b**) αS in the absence (grey) and presence of 50% POPG NDs (conditions analogous to data shown in Fig. S5, for 100% POPG NDs). (**c,d**,) Seeded aggregation assays under conditions analogous to data shown in Fig. S5, but using 50% POPG NDs. Surprisingly, the 50% charged NDs do show an effect on the ThT signal as a reporter for fibril elongation in seeded experiments. This data is difficult to explain given that elongation is, in all cases of amyloid formation, responsible for the generation of the bulk of fibril mass. Hence, its inhibition should slow down the overall aggregation kinetic, also under non-seeded conditions. Noteworthy, we observe that ThT signal intensity can be strongly affected by the presence of NDs and that the absolute ThT intensity does not correlate with absolute fibril mass when comparing data recorded in the absence or presence of NDs. This is visible by comparing SDS-PAGE results (**e**) with ThT intensities (**f**) of identical samples. After the aggregation assay the αS-band for soluble (sl) and insoluble (p) protein show very similar intensities in the absence and presence of 100% DMPC NDs. Although our NMR data show that these NDs do not interact with the αS, the corresponding ThT signal (of the identical samples) show very different intensities (f). It is therefore possible that the ThT (unspecifically) interacts with NDs, leading to an overall decrease in ThT intensities. Furthermore, it is possible that the attachment of ND to the growing fibrils interferes with efficient binding of ThT. We therefore normalized most ThT assays. Noteworthy, this effect is less pronounced for ND with 100% POPG (as e.g. visible in the data in Fig. S5), which could be due to the high coverage of the lipid surface by αS molecules that may reduce the unspecific ThT-ND interactions. In line with our other data recorded using 50% POPG NDs, albeit significantly reduced ThT sensitivity, only very moderate effects of the NDs on the fibril elongation process are visible after normalizing ThT intensities in the seeded aggregation assays (**h,i**).

αS type	pH	MSP type	Lipids in ND	αS-to-ND ratio	Used for data in
acetylated	5.3	-	-	-	Fig. S3c,e black
	7.4	-	-	-	Fig. S3a black
		D1	100% DMPC	2:1	Fig. 1a,b black
			25% POPG – 75% DMPC	2:1	Fig. 1a,b light blue
			50% POPG – 50% DMPC	1:1	Fig. 5g, yellow
				2:1	Fig. 1a,b blue<br>Fig. 5g, purple
				4:1	Fig. 5g, orange
				8:1	Fig. 5g, red
				16:1	Fig. 5g, blue
			75% POPG – 25% DMPC	2:1	Fig. 1a,b dark blue
		D5			Fig. S3a,b red
		D1	100% POPG	1:2	Not shown
				1:1	Fig. 5b, yellow
				2:1	Fig. 1a,b purple<br>Fig. 5b, purple<br>Fig. S3c,d purple
	5.3			2:1	Fig. S3c,d, orange
	7.4			4:1	Fig. 5b, orange
				8:1	Fig. 5b, red
				16:1	Fig. 5b, blue
			100% POPC	2:1	Fig. 3a, dark grey
			50% POPG – 50% POPC		Fig. 3a, yellow
			50% DMPG – 50% POPC		Fig. 3a, green
			50% DMPG – 50% DMPC		Fig. 3a, beige
			100% DMPG		Fig. 3a, brown
non-acetylated		-	-	-	Fig. S2a, grey
		D1	100% DMPC	2:1	Fig. 1c, black<br>Fig. S2a, black
			25% POPG – 75% DMPC	2:1	Fig. 1c, light blue<br>Fig. S2a, light blue
			50% POPG – 50% DMPC	1:1	Fig. S2h, yellow
				2:1	Fig. 1c, blue<br>Fig. S2a, blue<br>Fig. S2h, purple
				4:1	Fig. S2h, orange
				8:1	Fig. S2h, yellow
				16:1	Fig. S2h, blue
				32:1	Fig. S2h, dark blue
			75% POPG – 25% DMPC	2:1	Fig. 1c, dark blue<br>Fig. S2a, dark blue
			100% POPG	1:1	Fig. S2e, yellow
				2:1	Fig. 1c, purple<br>Fig. S2a, purple<br>Fig. S2e, purple
				4:1	Fig. S2e, orange
				8:1	Fig. S2e, red
				16:1	Fig. S2e, blue
				32:1	Fig. S2e, dark blue

Supplementary Table 1. Summary of NMR samples used in the study.

**Membrane models**	**Systems**	**αS initial distance to the membrane surface (nm)**	**Time (ns)**
POPC/POPG*	RUN1	-	500
DMPC/DMPG*	RUN2	-	1000
POPC/POPG	RUN3	0.5	1000
	RUN4	0.5	1000
	RUN5	1.5	1000
DMPC/DMPG	RUN6	0.5	1000
	RUN7	1.5	1000
	RUN8	1.5	1000

Supplementary Table 2. Summary of MD simulations used for the study.

Supplementary Note 1: Stability consideration of the lipid bilayer nanodiscs

While contradicting results on structural integrity of liposomes in the presence of αS have been reported, it seems that under conditions similar to our study, αS can rather quickly disrupt (POPG-based) vesicles or supported bilayers[2,3]. Therefore, it may be difficult in the obtained results to distinguish between effects that originate from interaction with (curved) intact bilayers or effects that are caused by lipid dissociation/ rearrangements. It can be expected that the presence of the scaffold protein protects the lipid bilayer to some extent from rearrangement and indeed our DSC data (Fig. S1e) suggest that the lipids in NDs are less affected by the presence of αS as compared to SUVs. This data, however, do not suffice to fully justify the assumption of an unchanging bilayer during the time course of the experiment. Noteworthy, it has also been reported that due to its amphipathic properties αS can encircle lipid bilayers in a similar way as the membrane scaffold protein and can form nanodiscs of slightly larger size[4,5]. Therefore, although thermodynamically unlikely, one cannot directly exclude the possibility that αS replaces the MSP to some extend during the experiments. However, as discussed in the following, our results strongly suggest that the NDs are stable during the time-course of the interaction experiments.

For moderately negatively charged nanodiscs (50% POPG) one strong indicator is the size exclusion chromatography data recorded after prolonged incubation (Fig. S1b). This data shows (in a quantitative way) that αS does not disrupt a detectable fraction of NDs within 24 h. Additionally the gradual increase of binding interface with increased charge density suggest that the recorded data report on α-Synuclein's interaction with the membrane surface (lipid head groups) and not on the 'encircling' of the hydrophobic acetyl chains. This aspects is valid for all NDs from 0-75% anionic lipid content.

For highly charged nanodiscs (100% POPG), resembling the 100% POPS used in[4] the interpretation of the SEC profile (see Fig. S1c) is more difficult. NDs and αS co-elute at a lower retention volume, this could either be explained by a replacement of the scaffold protein by αS in nanodiscs (forming larger discs, as reported in[4]) or by a decreased interaction with the SEC material arising from tightly bound αS molecules with a significant protruding region (residues 98 to 140). Our BLI measurements with immobilized 100% POPG NDs, however, show a clear full recovery of the signal after αS binding and release (Fig. 5a, Fig. S2d). Since only the MSP and not the αS is immobilized on the tip, this data show that during the time course of the experiment no disruption and/or MSP displacement and/or larger lipid rearrangement occur due to the presence of strongly interacting αS. Overall these data, in combination with the overall consistency of the data measured during the time course of several days, strongly indicates that the different experiments recorded on the different ND preparations report predominantly on the interaction with a stable planar bilayer.

While the stability assumption seems valid for all interaction studies, it is at this point not fully clear whether this also holds true for the aggregation assays. It has been shown that lipids from vesicles and reconstituted planar bilayers can be incorporated into fibrils during the aggregation process[3,6], which might be an important process for the toxicity of αS oligomers, forming pores in membranes[7,8]. Our AFM data do not show clear differences in morphologies (Fig. S6) as have been observed under similar conditions for aggregation assays in the presence of SUVs[1], suggesting that no/less lipids are incorporated into the αS fibrils. Native gels recorded after the aggregation assay do show intact NDs (data not shown), however denaturing gels after the aggregation also show some MSPs in the insoluble fraction, possibly indicating co-aggregation to some extent (Fig. S7e). In general, the consistency between the interaction studies and the visible effects in the aggregation assays suggest that the interaction with stable bilayers does have a considerable effect during the aggregation, however additional effects from lipid incorporation/rearrangements during the aggregation assays cannot be excluded.

Supplementary Note 2: Considerations regarding solution NMR detection of membrane-bound state

It is worth noting that the solution NMR results described in the manuscript refer to the decrease in peak intensity as a reporter for interactions, which is in line with the effects seen before using SUVs[17]. While it is clear that SUVs have particle sizes (associated with slow tumbling rates) well above the detection limit of conventional solution NMR techniques, the smaller size of the NDs system should, in principle, allow detection of NMR signals, as has been reported before for several ND-bound or ND-integrated proteins[18]. Nonetheless, neither the usage of Transverse Relaxation Optimized Spectroscopy (TROSY)[19] with increased signal accumulation (i.e. 10-fold longer as for spectra shown in Fig. 1a) nor the measurement at increased temperatures (35°C) and the usage of an NMR-optimized smaller membrane scaffold protein (MSP1D1ΔH5)[20] forming NDs of smaller size and higher tumbling rates, resulted in appearance of a new set of peaks indicative for the bound sate (and the presence of slow exchange processes) or a collective shift of peaks indicative of fast on-off exchange processes (Fig. S3).

In theory, three effects may explain this observation and obstruct detection of the ND-bound residues of αS: (i) the bound-to-free exchange rate is in the order of the NMR time scale (so-called intermediate exchange), (ii) the presence of a non-negligible part of αS protruding out of the ND (namely at least residues 98-140), slowing down molecular tumbling and increasing relaxation leading to line broadening beyond the detection limit, and/or (iii) membrane-bound αS shows a significant amount of plasticity leading to inhomogeneous broadening of the NMR lines.

While intermediate exchange can be largely ruled out due to the observed binding kinetics (BLI results, Fig. 5a, t_{on} and t_{off} of around 4 µs · M and 65 s, respectively) it is at this point difficult to further distinguish between slower tumbling and molecular plasticity (or a combination thereof) that interfere with solution NMR detection of the bound conformation.

Supplementary Note 3: Generation of structural models of aS nanodiscs interaction

Atomic coordinate files (.pdb-files) of nanodiscs with the appropriate lipids and scaffold protein were generated using the Charmm nanodiscs builder module[21]. αS with ideal α-helical secondary structure for regions interacting with the NDs was generated using the builder module in pymol (The PyMOL Molecular Graphics System, Version 1.7 Schrödinger, LLC.). The interacting regions reflect respective solution NMR attenuation profiles and secondary structure is in line with DNP solid-state NMR data. No distance restrains or additional structure calculation algorithms were used. The benefits of the resulting structural model are that it rather accurately reflects the respective molecular sizes, e.g. length of elongated helixes in respect to nanodisc diameter or molecular crowding on the nanodisc surface.

Supplementary References

1. Galvagnion, C. et al. Lipid vesicles trigger alpha-synuclein aggregation by stimulating primary nucleation. *Nat. Chem. Biol.* **11**, 229-234 (2015).
2. Fusco, G. et al. Structural basis of synaptic vesicle assembly promoted by alpha-synuclein. *Nat. Commun.* **7**, 12563; DOI:10.1038/ncomms12563 (2016).
3. Reynolds, N.P. et al. Mechanism of membrane interaction and disruption by alpha-synuclein. *J. Am. Chem. Soc.* **133**, 19366-19375 (2011).
4. Eichmann, C. et al. Preparation and characterization of stable alpha-synuclein lipoprotein particles. *J. Biol. Chem.* **291**, 8516-8527 (2016).
5. Eichmann, C., Kumari, P. & Riek, R. High-density lipoprotein-like particle formation of synuclein variants. *FEBS Lett.* **591**, 304-311 (2017).
6. Hellstrand, E., Nowacka, A., Topgaard, D., Linse, S. & Sparr, E. Membrane lipid co-aggregation with alpha-synuclein fibrils. *PLoS One* **8**, e77235; DOI:10.1371/journal.pone.0077235 (2013).
7. Auluck, P.K., Caraveo, G. & Lindquist, S. alpha-Synuclein: membrane interactions and toxicity in Parkinson's disease. *Annu. Rev. Cell Dev. Biol.* **26**, 211-233 (2010).
8. Butterfield, S.M. & Lashuel, H.A. Amyloidogenic protein-membrane interactions: mechanistic insight from model systems. *Angew. Chem. Int. Ed. Engl.* **49**, 5628-5654 (2010).
9. Maltsev, A.S., Ying, J. & Bax, A. Impact of N-terminal acetylation of alpha-synuclein on its random coil and lipid binding properties. *Biochem.* **51**, 5004-5013 (2012).
10. Dikiy, I. & Eliezer, D. N-terminal acetylation stabilizes N-terminal helicity in lipid- and micelle-bound alpha-synuclein and increases its affinity for physiological membranes. *J. Biol. Chem.* **289**, 3652-3665 (2014).
11. Bartels, T., Kim, N.C., Luth, E.S. & Selkoe, D.J. N-alpha-acetylation of α-aynuclein increases its helical folding propensity, GM1 binding specificity and resistance to aggregation. *PLoS ONE* **9**, e103727; DOI:10.1371/journal.pone.0103727 (2014).
12. Kang, L., Janowska, M.K., Moriarty, G.M. & Baum, J. Mechanistic insight into the relationship between N-terminal acetylation of α-synuclein and fibril formation rates by NMR and fluorescence. *PLoS ONE* **8**, e75018; DOI:10.1371/journal.pone.0075018 (2013).
13. Vacha, R., Linse, S. & Lund, M. Surface effects on aggregation kinetics of amyloidogenic peptides. *J. Am. Chem. Soc.* **136**, 11776-11782 (2014).
14. Campioni, S. et al. The presence of an air-water interface affects formation and elongation of alpha-synuclein fibrils. *J. Am. Chem. Soc.* **136**, 2866-2875 (2014).
15. Iyer, A. et al. The impact of N-terminal acetylation of alpha-synuclein on phospholipid membrane binding and fibril structure. *J. Biol. Chem.* **291**, 21110-21122 (2016).
16. Fusco, G. et al. Direct observation of the three regions in alpha-synuclein that determine its membrane-bound behaviour. *Nat. Commun.* **5**, 3827; DOI:10.1038/ncomms4827 (2014).
17. Bodner, C.R., Dobson, C.M. & Bax, A. Multiple tight phospholipid-binding modes of alpha-synuclein revealed by solution NMR spectroscopy. *J. Mol. Biol.* **390**, 775-790 (2009).
18. Viegas, A., Viennet, T. & Etzkorn, M. The power, pitfalls and potential of the nanodisc system for NMR-based studies. *Biol. Chem.* **397**, 1335-1354 (2016).
19. Pervushin, K., Riek, R., Wider, G. & Wuthrich, K. Attenuated T2 relaxation by mutual cancellation of dipole-dipole coupling and chemical shift anisotropy indicates an avenue to NMR structures of very large biological macromolecules in solution. *Proc. Natl. Acad. Sci. U. S. A.* **94**, 12366-12371 (1997).
20. Hagn, F., Etzkorn, M., Raschle, T. & Wagner, G. Optimized phospholipid bilayer nanodiscs facilitate high-resolution structure determination of membrane proteins. *J. Am. Chem. Soc.* **135**, 1919-1925 (2013).
21. Jo, S., Kim, T., Iyer, V.G. & Im, W. CHARMM-GUI: A Web-based Graphical User Interface for CHARMM. *J. Comput. Chem.* **29**:1859-1865 (2008).
